# Feasibility of a school-based peer-led high-intensity interval training intervention: the Young Fitness Leaders project

**DOI:** 10.1186/s12889-026-26543-w

**Published:** 2026-02-24

**Authors:** Kathryn L. Weston, Naomi L. Burn, Alexis Goroski, Matthew Weston, Brook Galna, Rosie Glossop, Maddey Patterson, Hannah Batten, Alfie Gordon, Laura Basterfield

**Affiliations:** 1 Department of Psychological Sciences and Health, University of Strathclyde, Glasgow, UK; 2 School of Applied Sciences, Edinburgh Napier University, Edinburgh, UK; 3 Adelaide University Online, Adelaide University, Adelaide, Australia; 4 Human Nutrition and Exercise Research Centre, Newcastle University, Newcastle Upon Tyne, UK; 5 Institute for Sport, Physical Education and Health Science, Moray House School of Education and Sport, University of Edinburgh, Edinburgh, UK; 6 Institute of Sport, Manchester Metropolitan University, Manchester, UK; 7 School of Allied Health (Exercise Science), Murdoch University, Murdoch, Australia; 8 Centre for Healthy Ageing, Health Futures Institute, Murdoch University, Murdoch, Australia; 9 Population Health Sciences Institute, Newcastle University, Newcastle Upon Tyne, UK

**Keywords:** Peer-leaders, High-intensity interval training, HIIT, Adolescents, Physical activity, Schools, Feasibility

## Abstract

**Introduction:**

While school-based high-intensity interval training (HIIT) has demonstrated efficacy for improving adolescents’ physical and mental health, interventions have largely been researcher-led, which limits scalability. This could be resolved via peer-led programmes, whereby older pupils (i.e., peer-leaders) deliver HIIT to younger pupils (i.e., peer-recipients). We aimed to explore the feasibility of a school-based peer-led HIIT intervention.

**Methods:**

Using a non-randomised controlled trial design, 44 Year 7 pupils (aged 12.1 ± 0.3 years [mean ± SD]) were recruited from one school in North East England, with 21 (8 girls) allocated as peer-recipients to an 8-week HIIT intervention, and 23 (17 girls) as controls. Five Year 12–13 pupils (aged 17.3 ± 0.4 years) were recruited as peer-leaders and received training on school-based HIIT based on boxing and whole-body exercises. Peer-leaders then delivered twice weekly HIIT sessions during morning tutor time. Primary outcomes were recruitment, retention, attendance and acceptability (explored in post-intervention focus groups with peer-recipients, peer-leaders and teachers). Secondary outcomes included intervention fidelity (via researcher field note observations and heart rate monitoring), and preliminary impact on physical fitness and psychological outcomes.

**Results:**

Recruitment was 72% (21/29 eligible pupils), 74% (23/31) and 42% (5/12) for peer-recipients, controls and peer-leaders, respectively. All control participants completed the study; one peer-leader left the school, and two peer-recipients withdrew (94% overall retention). Intervention attendance (expressed as percentage of scheduled sessions) was 73 ± 31%. The intervention was generally well-received by peer-recipients, peer-leaders and teachers. The mean peak heart rate across all repetitions was 76% of age-predicted maximal, with a between- and within-participant variability of 6% points and 8% points, respectively. For the physical fitness and psychological outcomes, intervention effect directions were inconsistent and effect estimates imprecise.

**Conclusions:**

Peer-led HIIT may represent a scalable and feasible school-based physical activity model from a recruitment, retention, attendance and acceptability perspective. However, intervention heart rates and session observations suggest HIIT activities were not always delivered and performed as intended, which could limit intervention effectiveness in larger-scale trials. Future iterations of peer-led HIIT programmes should focus on refining intervention delivery by providing an enhanced training and support package for peer-leaders.

**Trial registration:**

The trial was registered on ISRCTN, the UK’s Clinical Study Registry on 6th March 2023 (https://www.isrctn.com/ISRCTN89390191). The trial registration number is ISRCTN89390191.

**Supplementary Information:**

The online version contains supplementary material available at 10.1186/s12889-026-26543-w.

## Introduction

It is now well documented that the Covid-19 pandemic had a substantial impact on young people’s physical and mental health. For example, post-pandemic reductions in physical fitness, physical activity levels and mental well-being have been observed in the UK ([1, 2] and elsewhere (e.g. [3–5]; and have yet to fully recover [6]. Given the well-established protective roles that higher levels of physical fitness and physical activity play in current and future physical and mental health [7–9], it has been recommended that improving young people’s movement behaviours is at the forefront of pandemic recovery efforts [3]. This could be achieved through effective, evidence-based and engaging physical activity interventions [10], conducted in environments such as schools, which reach a large percentage of children and adolescents [11, 12].

Over the last decade, the effects of school-based high-intensity interval training (HIIT) interventions (i.e., intermittent bouts of activity performed above moderate intensity [13], alternated with periods of rest or low intensity active recovery [14]) have been extensively explored. Findings from several high-quality reviews and meta-analyses indicate that school-based HIIT, incorporating activities such as ball-sport drills, body-weight exercises, dance, boxing and running [10, 15], can be a potent method for improving various physical fitness, cardiometabolic health, mental health and cognitive outcomes in young people (e.g., [10, 16–21]. To date, however, most school-based HIIT interventions have been delivered by researchers, which limits scalability (i.e., the capacity of an efficacious intervention, delivered on a small scale or under controlled conditions to be expanded and reach a greater proportion of the eligible population, while retaining effectiveness [22]). The main exceptions are two large cluster randomised controlled trials conducted in Australia and New Zealand where teachers received training and support to deliver HIIT activities independently of researchers [23, 24]. Such an approach can increase the scalability and sustainability of an intervention, and it is now recommended that school-based HIIT programmes are designed with scale-up in mind [25]. Given adolescents’ preferences to interact with and possibly be influenced by their peers [26] and concerns over school teacher burden [27], a peer-led intervention represents a potentially scalable, but as yet unexplored, means of delivering school-based HIIT.

Peer-led interventions typically involve individuals who have volunteered to deliver support, information, guidance, or an intervention to their peers [28, 29], often in the role of a lay teacher [28]. Specifically, ‘peer-leaders’ describes those delivering an intervention to their peers, whereas ‘peer-recipients’ are those receiving an intervention from their peers [28, 30]. Within schools, the use of peer-led intervention strategies for promoting positive health behaviours, such as smoking cessation (e.g., [31]), healthy eating (e.g., [32]) and physical activity (e.g., [26]), is increasing [33]. For physical activity interventions specifically, recent reviews (e.g., [28, 29, 33, 34] indicate that peer-led interventions can be an efficacious way of improving physical activity behaviours and, in turn, associated health outcomes. There is also growing evidence that the benefits of peer-led interventions extend beyond just physical health [28, 34]; and can positively impact a range of academic, psychosocial and behavioural outcomes in both peer-recipients and peer-leaders [28]. To date however, few studies have explored the impact of peer-led physical activity interventions on cardiorespiratory and muscular fitness outcomes [28] and we are unaware of any school-based HIIT interventions which have used a peer-led delivery model as a means of increasing scalability. The aim of our study, therefore, was to explore the feasibility of a peer-led HIIT intervention, whereby older adolescents (i.e., peer-leaders) deliver HIIT sessions to younger pupils (i.e., peer-recipients) during the school day. Our primary objectives were to determine recruitment rate, retention and intervention attendance; and to explore peer-recipients’, peer-leaders’ and teachers’ experience and acceptability of the intervention. Our secondary objectives were to examine the fidelity of the intervention and the preliminary impact on physical fitness and psychological outcomes.

## Methods

### Pre-trial formative evaluation

Guided by our previous co-designed HIIT interventions (e.g., [35–37]) and recommendations on stakeholder engagement [38], we conducted a pre-trial formative evaluation in July 2022. This consisted of three focus groups with 17 younger school pupils (aged 11–15 years; representative of the intended target population for peer-recipients), one focus group with five older pupils (aged 16–17 years; representative of the intended target population for peer-leaders), a semi-structured interview with the physical education (PE) teacher assisting with the study co-ordination, and a short survey completed by 12 other teachers. The purpose was to utilise the collective feedback and ideas to enhance and refine our preliminary intervention plans [39]. Key insights which impacted our final intervention design are briefly summarised below; full details are in Supplementary File 1.

The main barriers to intervention participation in younger pupils were perceived mismatches between their exercise ability and peer-leader’s expectation/instruction, and awkwardness in front of older pupils. For the older pupils, intervention delivery concerns were being underprepared, feeling uncomfortable leading large groups, a lack of knowledge and experience in working with young people, and managing poor behaviour. Older pupils also thought that burpees and mountain climber exercises were not suitable for completing in school uniform. Collectively, this feedback helped inform the peer-leaders pre-intervention training (detailed in the Young Fitness Leaders training section) and the intervention activities (detailed in the HIIT intervention section and Supplementary File 2). Teacher feedback highlighted the importance of working with small numbers (i.e., classes from only one year group). This was reinforced by the co-ordinating PE teacher, who suggested involving only one class in the intervention, due to the logistics involved in trial organisation. Teachers also indicated the school morning break time (1030–1100 h) could be a suitable timeslot for the HIIT sessions. In October 2022, we presented our refined intervention design to the pupils and PE teacher for feedback and approval. No further suggestions were made at this stage.

### Study design

Our trial was registered at https://www.isrctn.com/ISRCTN89390191 on the 6^th^ March 2023, conducted in accordance with the Declaration of Helsinki, and Newcastle University’s Faculty of Medical Sciences Research Ethics Committee provided ethics approval (ref 2331/22078 and ref 2451/27290). Using the Consolidated Guidance for Behavioural Intervention Pilot and Feasibility Studies [38], our study was designated as a feasibility study which reflects the novel nature of the planned intervention delivery model (i.e., peer-led HIIT). As this model has not been utilised previously, a feasibility study was deemed most appropriate to collect essential information on the design, conduct, participant experience and preliminary impact of the intervention [40], which could then inform the planning of a larger-scale trial [38].

The study took place at the same site as the pre-trial formative evaluation; a secondary school in Ashington, North East England which is one of the 10% most deprived locations in England [41]. Based on the teachers’ feedback, we intended to cluster randomise participants from a single year group to the intervention or control condition. Unfortunately, school timetabling constraints necessitated that the selection of Year 7 school year group and the grouping of two classes (i.e., intervention or control), drawn from the eight across the Year 7 school group, was made by the co-ordinating PE teacher, not at random. This meant we had to adopt a non-randomised controlled trial design, which is a deviation from our registered protocol. Accordingly, the design, conduct and reporting of our trial adheres to the Transparent Reporting of Evaluations with Non-randomised Designs statement [42] (Supplementary File 3).

### Trial recruitment

Recruitment took place in March 2023. Our target sample size was 70, consisting of 60 Year 7 pupils (reflective of the total number of pupils in two Year 7 classes combined) and 10 Year 12–13 pupils. For the Year 12–13 pupils, our target sample size of 10 was to enable eventual peer-leaders to deliver HIIT sessions on a rota system.

Across the two Year 7 classes, 60 pupils (aged 11 to 12 years) were invited to take part. Pupils from one class (*n = *29) were invited to join the peer-led HIIT intervention group; those in the second class (*n* = 31) were invited to join the control group. This was conducted through an in-school recruitment session, where all attending pupils received an information pack containing a parent/guardian information sheet, a Physical Activity Readiness Questionnaire (PARQ) and an opt-out form. For the recruitment of peer-leaders (referred to as Young Fitness Leaders herein), Year 12–13 pupils (aged 17 to 18 years) were invited to a separate in-school recruitment session. Of the 12 pupils that attended, two were Year 13 pupils who had taken part in the pre-trial focus groups in 2022. For all participants, study exclusion criteria were the presence of conditions or injuries that stop participation in high-intensity exercise; diabetes mellitus; heart or vascular complaints; early family history of sudden death, and pregnancy or likelihood of pregnancy. Year 7 and Year 12–13 pupils were eligible for enrolment if they were free from the study exclusion criteria (determined via the completion and return of the PARQ) and provided written participant informed assent. On the advice of the school, we gained ethical approval for opt-out parent/carer consent. This negated the requirement for additional signed parent/carer consent and assumed consent unless the parent/carer actively opted their child out by returning the opt-form provided. Forty-four Year 7 pupils (21 intervention group) and five Year 12–13 pupils provided participant assent and were free of study exclusion criteria. To our knowledge, none of the five Year 12–13 pupils held leadership roles within the school (e.g., Head Girl/Boy, School Captain positions etc.), although two assisted with youth football coaching at external clubs.

### Intervention overview

Our peer-led HIIT intervention is described according to the requirements of the Template for Intervention Description and Replication (TIDiER) checklist [43] (Supplementary File 4) and the Consensus on Exercise Reporting Template (CERT; [44]) (Supplementary File 5). The intervention took place over nine calendar weeks from April to June 2023, which incorporated the 8-week intervention, and a 1-week mid-term school holiday after intervention week 5. For intervention participants (referred to as peer-recipients herein), the peer-led HIIT sessions were completed in addition to their usual school PE lessons and physical activity. Control participants continued their usual school routine, PE lessons and physical activity throughout the intervention period and did not receive any supplementary activities.

### Young Fitness Leaders training

One week prior to the intervention start, the five Young Fitness Leaders (three female) received a 1 h in-person training session on the delivery of HIIT. The session was led by a researcher who has extensive experience in the design and delivery of school-based HIIT programmes and supported by three other researchers. The in-person training was supplemented with a 34-page training manual (shown in Supplementary File 2) which was given to all the Young Fitness Leaders. The training manual contents were guided by our formative evaluation, previous research on the successful delivery of school-based HIIT, and the SAAFE (Supportive, Active, Autonomous, Fair, Enjoyable) teaching principles [45]. Briefly, the manual included a simple definition of HIIT and details of the Young Fitness Leaders’ role in the HIIT sessions, including an illustrative diagram of the HIIT protocol they would deliver. Specifically, our HIIT protocol required recipients to perform four repetitions of 45 s maximal effort exercise, each interspersed with 60 s rest. The HIIT repetitions were based on body-weight exercises and non-contact boxing, which have demonstrated efficacy in inducing physiological responses indicative of high-intensity work in young people (e.g., [23, 25, 35]), have been well-received in previous school-based HIIT interventions (e.g., [46, 47]) and reflect some of the suggestions from our formative evaluation. Twenty pages of the training manual provided instructions and images on how to perform and progress different types of body-weight and boxing-style HIIT (e.g., star jumps, squat jumps and boxing actions; detailed examples shown in Supplementary File 2), similar to the pictorial HIIT cards created to support the delivery of a teacher-led HIIT programme in Australia [23]. The manual concluded with session planning and Young Fitness Leader self-reflection templates (detailed further in subsequent sections).

The training session was structured around the manual, such that each aspect was discussed amongst the group, then followed by a practical activity. For example, Young Fitness Leaders trialled some of the HIIT activities in the manual, first as a participant and then as a peer-leader instructing the rest of the group. To address behaviour management and peer-recipient engagement concerns raised by older pupils in the formative evaluation, we utilised role play activities to act out different scenarios (e.g., disinterested and/or disruptive pupils), then discussed potential solutions as a group. We also highlighted that managing pupil behaviour issues, injuries etc. were the responsibility of the supporting school staff members and researchers. Using the manual session planning templates, we discussed how to plan and prepare for the HIIT sessions. To increase the likelihood of positive interactions between the Young Fitness Leaders and peer-recipients and minimise potential mismatches in expectation/instruction and exercise ability, we also provided verbal and written guidance on gauging exercise intensity, peer-leader qualities, and discussed the SAAFE principles [45] in the context of delivering HIIT. For example, we reinforced the importance of creating a supportive and enjoyable environment for the peer-recipients, by enabling autonomy [48] with HIIT activities and partner selections where possible. We also cautioned against delivering sessions in ways which could create an unsupportive or controlling environment, and lead to recipients feeling judged and under pressure to perform [48, 49] (e.g., selective praise, setting unrealistic exercise targets and being dismissive of recipients’ feedback/concerns). Finally, we explained the purpose of the self-reflection template in the manual, which Young Fitness Leaders were asked to complete on a weekly basis. Young Fitness Leaders were also given access to online training materials, which were hosted on a secure virtual learning environment and intended to supplement the in-person training and manual. Here, different sections of the manual were delivered as short videos with voiceovers. Video demonstrations of the HIIT activities shown in the manual were also provided.

During the training session, the Young Fitness Leaders indicated a preference for beginning the intervention with activities they were already familiar with. As such, it was agreed that the first three weeks of the HIIT sessions would focus on body-weight exercises. To further prepare the Young Fitness Leaders for delivering non-contact boxing activities, one research team member led a refresher training session focused exclusively on boxing activities. This took place at the start of week 4, the day before the scheduled HIIT sessions for that week.

### HIIT intervention

Peer-led HIIT sessions were designed and facilitated by the Young Fitness Leaders, with multiple leaders involved in each session. Sessions were observed by at least two researchers and a school staff member. Peer-recipients and Young Fitness Leaders were encouraged to attend as many sessions as possible, with the latter also expected to undertake a pre-designated delivery role during each session (e.g., take the warm-up and/or HIIT activities, oversee and communicate work:rest timings for HIIT repetitions etc.). Details of the HIIT protocol are shown in Table 1.

**Table 1:** HIIT protocol characteristics

Training Characteristic	Details
Session Frequency	Twice weekly (Tuesdays & Thursdays)
Session Timing	08:30–08:45 (School morning tutor time)
Session Location	Indoor school hall (Weeks 1—3) and Outdoor school courtyard (Week 4—8)
Session delivery	Small groups of 4—6 recipients, each led by one Young Fitness Leader
Warm-Up	90—120 s of whole-body movements relevant to the session
HIIT repetitions (number and length)	Four 45-s repetitions
Rest period between repetitions	60 s
Intensity Descriptor	Recipients were verbally encouraged to provide a maximal effort. The HIIT criterion was ≥ 85% of age-predicted maximum heart rate
Progression across 8-week intervention	HIIT repetition length increased by 5 s every fortnight
Exercises	Whole‑body bodyweight exercises from the training manual (Weeks 1—3) and non-contact boxing drills (Weeks 4—8)
Equipment	No equipment required for body-weight exercises
	Boxing drills: Boxing gloves and focus pads (donated by Newcastle University)

Shown in Table 1, our session time of 0830–0845 h (i.e., during school morning tutor time) deviated from that suggested in the formative evaluation (i.e., 1030–1100; school morning break time). This was necessitated due to logistical concerns raised by the school management team. During HIIT sessions therefore, control participants attended morning tutor time as normal, which involved school registration and sitting in a classroom with their form tutor and classmates for 15 min. Further, as detailed in our trial registration, we had prospectively intended to include a third teacher-led HIIT session during peer-recipients' weekly PE lessons. Due to staffing issues within the school PE department, however, this was no longer possible.

### Primary outcome measures

#### Recruitment, retention and intervention attendance

Recruitment data were collected by recording the number of Year 7 and Year 12–13 pupils who received study information and comparing this to the number that provided individual assent for participation and were free from study exclusion criteria. Retention describes the number of recruited pupils (i.e., Year 7 and Year 12–13 pupils) who were still involved at the end of the study. Intervention attendance was recorded via a register at each session and reported as a percentage of the total sessions scheduled (*n* = 16).

#### Programme experience and acceptability

Within two weeks of the final HIIT session, the Young Fitness Leaders and all peer-recipients who completed the 8-week intervention were invited to take part in a focus group to discuss their experiences of being part of the trial. In addition, two semi-structured interviews were conducted with two teachers. The focus group and semi-structured interview scripts (Supplementary File 6) were devised by two researchers; then shared with a third researcher for feedback. All focus groups and interviews were audio recorded and transcribed verbatim by one researcher, then checked for completeness and accuracy by a second researcher.

In addition to the focus group, Young Fitness Leaders were also asked to submit a short individual self-reflection on the HIIT sessions they had delivered on a weekly basis throughout the intervention period. As detailed in the training manual, we requested that reflections were based on how they thought sessions had gone that week, whether anything could be improved for the following week, and what they thought had worked well.

##### Peer-recipient focus groups

Two peer-recipient focus groups were conducted in July 2023. Questions focused on peer-recipients’ experiences of taking part in the HIIT programme; in relation to: Hhow the HIIT sessions were ran; how they felt during the HIIT sessions; their perspectives on the Young Fitness Leaders running the sessions; and suggested changes for future iterations of the programme. The focus groups were led by two researchers who had not been present at the peer-led HIIT sessions.

##### Young Fitness Leaders focus groups and weekly self-reflections

One focus group took place with Young Fitness Leaders in July 2023. One Young Fitness Leader was absent on the day, so took part in a rescheduled one-to-one interview which followed the same script and structure. Questions focused on Young Fitness Leaders’ experiences of delivering the HIIT intervention, in relation to: the pre-intervention training; delivering the HIIT sessions; perceptions on peer-recipients’ experience and engagement; suggested changes for future iterations of the intervention; and the overall Young Fitness Leader experience. The focus group was led by a researcher who had not been present at the peer-led HIIT sessions or the Young Fitness Leaders training session. Due to rescheduling logistics, the one-to-one interview with the Young Fitness was conducted by the lead researcher who had attended the peer-led HIIT sessions and the Young Fitness Leaders training session.

For the weekly self-reflection task, one female Young Fitness Leader submitted seven of a possible eight reflections. Another female submitted six reflections, and both males did not submit any reflections.

##### School teacher semi-structured interviews

Two semi-structured interviews were conducted; with the co-ordinating PE teacher and with the Year 7 form teacher (the teacher with twice-daily contact time with peer-recipients). These were conducted in-person by the lead researcher. Questions focused on the teachers’ perceptions of the delivery and impact of the study, in relation to those directly involved in the study (e.g., peer-recipients and Young Fitness Leaders) and the wider school community. Teachers were also asked to share their perspectives on future iterations of the programme.

### Primary outcomes data analysis

#### Recruitment, retention and intervention attendance

Study recruitment and retention and intervention attendance, dose and overall time commitment are descriptively reported in raw units and percentages where appropriate.

#### Programme experience and acceptability

Following transcription, there were 66 pages of raw data (Arial font size 12, single line spacing) (21 pages from peer-recipients; 32 from Young Fitness Leaders and 13 from School Teachers). Focus group and interview data from each distinct group (i.e., peer-recipients, Young Fitness Leaders and teachers) were analysed separately. For the Young Fitness Leaders only, the individual submitted self-reflections were merged with focus group/interview data.

We used NVivo14 to conduct inductive thematic analysis [50].The following steps were conducted by one researcher who had not attended the Young Fitness Leaders training, any of peer-led HIIT sessions or the pre- or post-intervention measurement sessions. All transcripts were read and re-read to enable familiarisation with the data. Transcripts were then re-read line by line and marked with initial codes that described the content. Following discussions on the initial coding process, the researcher developed themes within the data and drafted initial data themes. Two researchers then reviewed and refined the themes using an iterative process, until agreement on the themes was reached and the final themes named. Finally, the themes were presented in a coherent and logical way alongside quotes deemed to best illustrate each theme [50].

### Secondary outcome measures

#### Intervention fidelity

In line with other school-based HIIT studies which have utilised non-researcher delivery models (e.g., [47]), we assessed the fidelity of our intervention using several metrics. To explore the degree to which Young Fitness Leaders delivered the intervention as intended and the quality of the delivery [51, 52], the lead researcher observed the HIIT sessions and collated written field notes (detailed below). The Young Fitness Leaders were also encouraged to reflect on their role as HIIT delivery agents via the weekly self-reflection task and in the post-intervention focus group, as detailed previously. This enabled us to gain insights on the acceptability of the trial from Young Fitness Leaders’ perspective, and aspects which may have impacted the fidelity of their delivery (e.g., the pre-intervention training, confidence in intervention delivery, and any factors which impacted their ability to deliver the HIIT sessions as planned [53]). To examine the extent to which peer-recipients performed the intervention as intended [54]), we monitored their heart rate responses during the HIIT sessions (detailed further below).

##### Researcher field note observations

Field notes were recorded by the lead researcher for 15 of the 16 HIIT sessions delivered across the intervention. One session was not observed due to illness. The notes contained in-depth descriptions of the following; Young Fitness Leaders attendance, roles assigned to each leader, session preparation and delivery, activities delivered, adherence to work:rest timings, and any other relevant and important information on the study context (e.g., reporting on the intervention environment and interactions with the peer-recipients) [55]. To encourage fidelity, [38] and facilitate reinforcement of key aspects of the pre-intervention training when necessary, field note observations were collated at the end of each intervention week to inform the contents of a supportive feedback email which was sent to the Young Fitness Leaders.

##### Intervention heart rate monitoring

Across the 8-week intervention, participants’ heart rate responses were recorded via wrist-worn monitors (Polar Unite, Polar Electro, Finland). On average, this occurred once per week for each participant (i.e., half the intervention group wore monitors for the first session of the week, and the other half for the second session). This was due to the total number of peer-recipients (*n* = 21) being higher than the maximum number of heart rate monitors available (*n* = 10). For each participant, age-predicted maximal heart rate (HR_max_) was calculated using the Tanaka Eq. (208—0.7 ∗ age in years) [56]. If a participant exceeded this predicted value during a HIIT session, their HR_max_ was recalibrated to the higher observed value [57]. Following each session, individual participant heart rate files were downloaded into the Polar Flow software (Polar Electro, Kempele, Finland). The data used in the statistical analysis was the highest 1 s value from each HIIT repetition, expressed as a percentage of the individual participant’s HR_max_, across each attended HIIT session [37]. In line with previous investigations showing a beneficial effect of HIIT, the target heart rate for high-intensity exercise was set at ≥ 85% age-predicted HR_max_ [58]. As detailed in our trial registration, we also intended to collect peer-recipients’ session Ratings of Perceived Exertion (RPE) as a means of quantifying exercise intensity using a potentially more cost effective and scalable tool than heart rate monitors [59]. Unfortunately, during the intervention the RPE data collection procedures fell below those that would be expected for reliable and robust RPE data. For example, the reduced data collection time necessitated by the intervention being moved to the short morning tutor time slot (15 min), made adherence to recently published guidelines for collecting RPE in applied environments challenging [60]. Consequently, we did not include the RPE data in analyses.

#### Physical fitness and psychological outcome measures

Baseline testing of the below secondary outcome measures took place the week before the intervention started. Post-intervention assessments were conducted up to seven days after the intervention ended. Outcomes were assessed in all participants (i.e., peer-recipients, control group participants and Young Fitness Leaders) unless otherwise stated. All testing was conducted indoors in the hard floor school sports hall by trained assessors who were not blinded to the group condition of the participants. The exception to this was post-intervention testing of 20 m Shuttle Run Test Performance (20mSRT), which took place outside due to exams being held the school hall. As detailed in our trial registration, we also intended to use device-based measures of physical activity to explore physical activity behaviours of our participants. Unfortunately, due to issues such as participants not returning their accelerometers, very poor compliance with wear-time criteria and non-completion of physical activity diaries intended to accompany the accelerometer data, only 16% of participants completed this aspect of the study. Due to concerns over the potential bias introduced due to missing data, we elected not to analyse this data.

#### Physical fitness

Physical fitness was measured using the following components of the Youth Fitness International Test (YFIT) Battery [61] and the Eurofit testing battery [62]; body mass index (BMI), 20mSRT performance, handgrip strength, standing broad jump (YFIT battery) and sit-and-reach performance (Eurofit battery). Test procedures have been published elsewhere (e.g., [1, 63]) and are summarised below.

##### Anthropometry

Participants’ body mass, standing height, and sitting height were measured to the nearest 0.1 kg and 0.1 cm using calibrated scales (Shekel H151-7, Shekel Scales LTD; Lower Galilee, Israel) and a portable stadiometer (Leicester Height Measure, SECA UK LTD, Birmingham, England), respectively. Two measurements were taken for each variable, then averaged for analysis. During assessments, participants wore light indoor clothing and were barefoot. Participants’ BMI (kg/m^2^) and BMI z-scores (age- and sex-specific) were calculated using the freely available LMSgrowth Excel add-in [64] relative to UK 1990 reference data [65]. UK population-sensitive cut-offs categorised participants as either “underweight” (≤ 2nd centile or below), “healthy weight” (> 2nd < 85th centile) “overweight or obesity” (≥ 85th centile) [66]. Participants’ leg length was calculated by subtracting sitting height from stature. Somatic maturity was estimated for each participant by predicting years from attainment of peak height velocity (i.e., maturity offset) via sex-specific multivariable equations [67].

##### 20 m Shuttle run test performance

Cardiorespiratory fitness was indirectly assessed via 20mSRT performance using the British National Coaching Foundation protocol [68]. Participants were encouraged to run between cones in time with an audible bleep signal for as long as possible and were asked to stop if they failed to maintain the specified pace for two consecutive shuttles. Participants were also allowed to drop out at their own volition at any time if they felt unable to maintain the required pace. Test performance was expressed as the number of shuttles completed.

##### Handgrip strength

Upper body muscle strength was assessed via handgrip strength, using a digital hand dynamometer (Grip-D, TKK 5401, Takei, Tokyo, Japan). Participants stood with the wrist in a neutral orientation and elbow of the testing arm completely extended and without touching any other part of the body [69], then squeezed the dynamometer as hard as they could continuously for at least 3 s. Elbow flexion from 180° to 90° was permitted [70]. Following a recovery period of at least 3 min, the test was repeated. The maximum score for each hand (in kg) was recorded for analysis.

##### Standing broad jump

Lower body muscular power was measured via standing broad jump performance. Participants were instructed to stand behind a starting line in the sports hall and, with their feet together, jump forward as far as possible. Participants performed three practice jumps, followed by three measured attempts. The distance jumped was measured from the starting line to where the back of the heel nearest to the starting line landed [71] using a tape measure, with the maximum score (recorded in cm) retained for analysis.

##### Sit-and-reach performance

Flexibility was assessed using the sit-and-reach test, using a steel sit-and-reach box. Participants were required to sit on the sports hall floor with their legs straight and feet against the box. They were then asked to reach out with both hands as far as possible. Three attempts were permitted with the maximum score (recorded as the last complete 0.5 cm) retained for analysis.

#### Psychological outcomes

##### Health-related quality of life (HRQoL)

Participants’ HRQoL was measured via the Kidscreen-27 [72] questionnaire, validated to assess subjective health and wellbeing in children and adolescents aged 8–18 years. It has 27 items measuring five dimensions: Physical Wellbeing; Psychological Wellbeing, Parent Relations & Autonomy, Social Support & Peers; School Environment; at least 75% of items had to be answered for the questionnaire to be considered valid. Within each dimension, item scores are summed and transformed to T-scores with a mean ≈ 50 and SD ≈ 10. Higher scores indicate a higher HRQoL [72].

##### Sports club participation

Sports club participation was measured through a modified Leisure Time Physical Activity Survey [73]. Participants provided details on whether they attended sports clubs at school and outside of school and the type, weekly frequency and duration of club. Total time spent in sports clubs per week was calculated.

##### Self-efficacy for physical activity (Year 7 participants only)

Participants’ self-efficacy for being physically active was measured using the Physical Activity Self-Efficacy Scale [74]; a validated 8-item questionnaire which includes three subscales (barriers, support seeking, and positive alternatives). Responses are given on a 3-point Likert-type scale (no; not sure; yes), with a minimum and maximum score of 8 and 24, respectively.

##### Physical activity enjoyment (Year 7 participants only)

Participants’ physical activity enjoyment was measured via the Short Version of the Physical Activity Enjoyment Scale [75]. This validated 4-item questionnaire assesses feelings about physical activity via a 5-point Likert scale (strongly disagree to strongly agree), with a minimum and maximum score of 4 and 20, respectively.

##### Coaching efficacy scale (Young Fitness Leaders only)

Young Fitness Leaders’ coaching efficacy was assessed using the Coaching Efficacy Scale [76]. Questions began with the stem “How confident are you in your ability to…” followed by 17 items, with a 0 (not at all confident) to 9 (extremely confident) scale for responses. As this questionnaire was designed for adult sports coaches, questions were adapted where applicable to make them suitable, e.g. changing “Help athletes maintain confidence in themselves” to “Help your Year 7 group maintain confidence in themselves”. Questions on game strategy were inapplicable and not included.

### Secondary outcomes data analysis

#### Intervention fidelity: researcher field notes

The lead researcher’s field notes from each HIIT session were collated into a word document, analysed and summarised by one researcher who had not attended the Young Fitness Leaders training, any of the peer-led HIIT sessions or the pre- or post-intervention measurement sessions. Data were analysed using inductive thematic analysis [50], using the same process as outlined for the focus groups with peer-recipients and Young Fitness Leaders.

#### Intervention fidelity: intervention heart rate data

All visualisations and analyses were performed in R (version 4.1.2, R Foundation for Statistical Computing). For the participants wearing heart rate monitors during the HIIT sessions, there were no missing heart rate data, giving a total of 508 measured repetitions across the intervention. We calculated the proportion of repetitions meeting our HIIT criterion, along with the 95% confidence interval (95%CI) for this proportion obtained via the PropCIs package [77]. Heart rate data, categorised as meeting or not meeting the HIIT criterion, along with the individual data points, median, boxplot, and density plot are visualised via raincloud plots [78], created using the ggrain package [79]. A linear mixed model obtained the estimate and precision (expressed as a 95% confidence interval [95%CI]) of the study heart rate data along with the between- and within-participant variability (expressed as SD’s) [80]. Repetition was modelled as a fixed effect [81] with crossed random effects for participant and session, and the estimation of variance components, i.e., the proportion of the total variability attributable to each random effect, calculated via the mixedup package [82]. Despite heart rate being a bounded variable, model residuals showed no evidence of heteroscedasticity.

#### Physical fitness, and psychological outcome measures

##### Year 7 participants (i.e., peer-recipients and control participants)

All visualisations and analyses were performed in R (version 4.1.2, R Foundation for Statistical Computing). Pre- and post-intervention outcome measures (physical fitness and psychological outcomes) are also visualised by raincloud plots. Analysis of covariance (ANCOVA) compared intervention effects with group (intervention, control) as the independent variable and change score (variable post-score minus variable pre-score) as the dependent variable. To control for any baseline imbalances, variable baseline values were used as covariates in the analysis [83] with additional covariates of sex and maturity offset for physical fitness measures and sex for psychological outcomes. Intervention effects are presented as the ANCOVA adjusted mean between-group difference with uncertainty expressed as 95% confidence intervals (95%CI). Cases with missing post-intervention data (*n* = 9 for physical fitness measures [4 intervention, 5 control], *n* = 7 for psychological measures [4 intervention, 3 control]) were removed from the analysis as missing post-intervention data contribute no information regarding the intervention effect [35]. Participants with post-intervention data but missing baseline data were included as these data contribute information about intervention effects and should be included in the analysis according to the intention-to-treat principle [35]. For the physical fitness measures, there was one participant with missing baseline scores for the 20 m SRT and standing broad jump and one participant with a missing 20 m SRT score – both participants were in the control group. For the psychological measures, there was one participant in the intervention group with missing self-efficacy and enjoyment scores. As per the recommendations [84], missing data were imputed using multiple imputation (method = predictive mean matching) performed via the mice package [85] with the pooled model results reported for 20 m SRT, standing broad jump, self-efficacy and enjoyment.

##### Young Fitness Leaders

Given the small sample size of the Young Fitness Leaders group at baseline (*n* = 5) and two Young Fitness Leaders being unable to the complete the post-intervention testing (reasons detailed in the results section), it was deemed unsuitable to analyse and report group level responses in the Young Fitness Leaders cohort. Due to concerns over potentially identifiable data, we also elected not to report individual responses.

## Results

### Primary outcomes

### Recruitment, retention and intervention attendance

#### Recruitment

##### Year 7 pupils

**Fig. 1:**
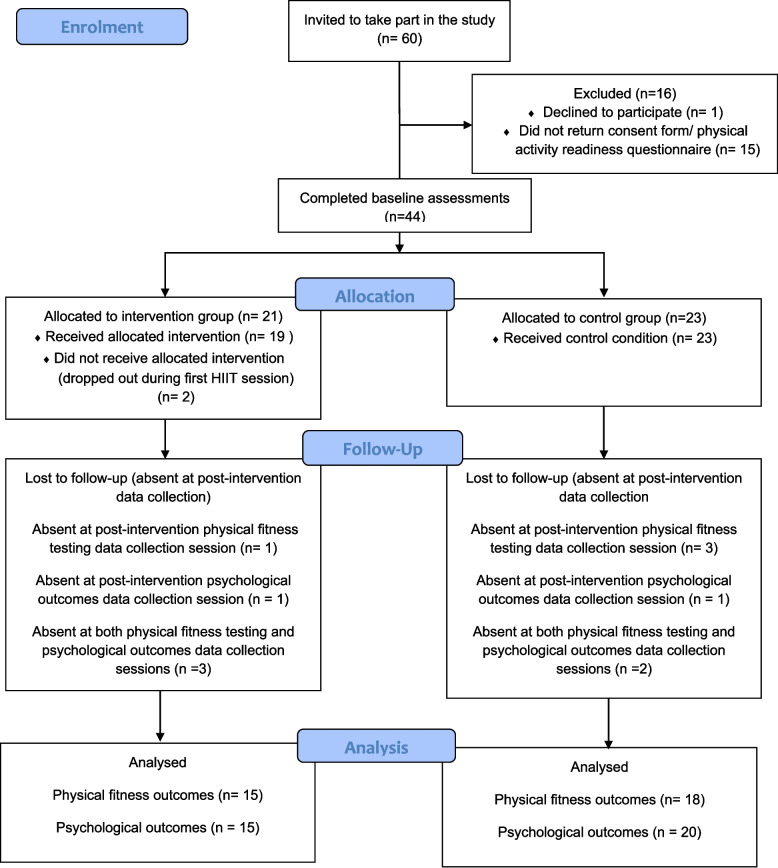
Year 7 participants’ flow through the study

Year 7 participant flow through the study is shown in Fig. 1. In the control group, 23 (six boys and 17 girls; aged 12.2 ± 0.3 years [mean ± SD]) of the 31 invited pupils enrolled into the study (74% recruitment rate), and in the intervention (i.e., peer-recipient) condition, 21 (13 boys and eight girls; aged 12.1 ± 0.4 years) of the 29 invited pupils enrolled (72%). Descriptive data of the Year 7 participants’ baseline characteristics are shown in Table 2.

##### Young Fitness Leaders

Of the twelve Year 12–13 pupils that attended the peer-leaders recruitment session, five (42%, two male and three females; mean age ± SD 17.3 ± 0.4 years) went on to become Young Fitness Leaders. Descriptive data of the Young Fitness Leaders’ baseline characteristics are shown in Table 3.

#### Retention

As shown in Fig. 1, two intervention participants withdrew from the trial during the first HIIT session, citing disinterest. Nineteen participants completed the intervention (90% retention), but three were absent at both post-intervention data collection sessions for the physical fitness and psychological outcomes. One participant was absent for the post-intervention physical fitness testing only, and one participant missed the post-intervention data collection session for the psychological outcomes. There were no dropouts in the control group (100% retention), but two were absent at both post-intervention data collection sessions for the physical fitness and psychological outcomes. Three participants were absent for the post-intervention physical fitness test only, and one participant missed the post-intervention data collection session for the psychological outcomes. In the Young Fitness Leaders group, one participant moved schools after intervention week 5 and was therefore unable to complete any further aspects of the study (80% retention). One Young Fitness Leader sustained a knee injury outside of the intervention, so was unable to complete the post-intervention physical fitness tests.

#### Attendance

Mean intervention attendance (expressed as percentage of scheduled sessions) for peer-recipients was 73 ± 31%, inclusive of the two peer-recipients who withdrew following the first HIIT session. For the four Young Fitness Leaders who completed the intervention delivery, mean attendance ± SD was 86 ± 16%. No intervention-related injuries or adverse events occurred during the trial.

**Table 2:** Year 7 participants baseline characteristics. Data summarised as means ± SD unless otherwise stated

Variable	Control (*n* = 23)	Intervention (*n* = 21)
Sex (male/female)	6/17	13/8
Age (years)	12.2 ± 0.3	12.1 ± 0.4
Maturity offset (years)	−0.1 ± 1.1	−1.1 ± 1.2
Height (cm)	155.4 ± 8.2	152.0 ± 8.1
Weight (kg)	55.3 ± 18.8	47.3 ± 12.3
BMI z score	1.09 ± 1.43	0.69 ± 1.19
Weight category		
Healthy weight (n, %)	10, 44	13, 62
Overweight and obesity (n, %)	13, 56	8, 38
Ethnicity White British (n, %)	21, 91	21, 100
20mSRT performance (shuttles)	21 ± 12	31 ± 17
Standing Broad Jump (cm)	124 ± 22	133 ± 28
Left hand grip strength (kg)	19.0 ± 5.2	17.3 ± 3.8
Right hand grip strength (kg)	20.0 ± 5.9	18.9 ± 3.9
Sit-and-reach (cm)	16.1 ± 10.0	13.6 ± 9.0
Total sports club time (min/wk)	401.5 ± 473.2	224.4 ± 198.7
HRQoL: Physical Wellbeing	48.2 ± 9.2	48.7 ± 10.5
HRQoL: Psychological wellbeing	49.6 ± 9.7	49.1 ± 10.8
HRQoL: Parent relations and autonomy	55.9 ± 11.5	50.2 ± 8.6
HRQoL: Social support and peers	52.2 ± 9.4	53.3 ± 13.1
HRQoL: School environment	50.2 ± 8.6	41.2 ± 10.3
Self-efficacy for physical activity	21.0 ± 2.6	20.9 ± 4.0
Physical activity enjoyment score	15.9 ± 3.8	16.7 ± 4.5

*BMI* Body Mass Index, *20mSRT* 20 m shuttle run test, *HRQoL* Health-related Quality of Life

**Table 3:** Young Fitness Leaders’ baseline characteristics Data summarised as means ± SD unless otherwise stated

Variable	Young Fitness Leaders (n = 5)
Sex (male/female)	2/3
Age (years)	17.3 ± 0.4
Height (cm)	173.4 ± 10.6
Weight (kg)	81.8 ± 16.5
BMI z score	1.61 ± 1.32
Weight category	
Healthy weight (n, %)	2, 40
Overweight and obesity (n, %)	3, 60
Ethnicity White British (n, %)	5, 100
20mSRT performance (shuttles)	36 ± 27
Standing Broad Jump (cm)	167 ± 36
Left hand grip strength (kg)	31.8 ± 7.3
Right hand grip strength (kg)	35.1 ± 6.5
Sit-and-reach (cm)	16.7 ± 7.9
Total sports club time (min/wk)	192 ± 217
HRQoL: Physical Wellbeing	40.1 ± 2.6
HRQoL: Psychological wellbeing	44.4 ± 10.0
HRQoL: Parent relations and autonomy	46.6 ± 8.5
HRQoL: Social support and peers	39.2 ± 3.3
HRQoL: School environment	44.5 ± 6.2
Coaching Self-efficacy	120.2 ± 22.9

*BMI* Body Mass Index, *20mSRT* 20 m shuttle run test, *HRQoL* Health-related Quality of Life

### Programme experience and acceptability

#### Peer-recipients’ perspectives

Three themes were identified regarding the peer-recipients’ experiences. “Wake-up woes” describes the impact of the early morning sessions on the acceptability of HIIT, while “Moving beyond repetition” highlights the demand for a wider variety of activity modes. Lastly, “Navigating intense exercise through peer-support” centred around the role of the Young Fitness Leaders in fostering a relatable and motivating environment.

##### Wake up woes

Opinions varied between peer-recipients on how they felt about the HIIT sessions. Whilst some said the sessions were “*Fun, yeah, really fun, yeah*”, others felt “*Lazy*”, “*Dead*”, or “*Tired*” owing to the sessions being in the morning. The main issue identified by the peer-recipients was the timing of the sessions being too early, which impacted them in various ways:

*“It was early in the morning and I was tired.”* (Female participant 1, Group 1)

[Would prefer to] *“Do them [*the HIIT sessions*] in the afternoon because we have to, like wake up*. [In the morning] *We have to just go straight there and sometimes, like, my bus is really late so I'm just there when everyone else is leaving.”* (Male participant 1, Group 2)

*“When it finished, you would go to your lesson* [late], *and all your teachers would always ask where you had been”* (Female participant 2, Group 1)

Some female peer-recipients expressed discomfort from being hot during the sessions and then remaining sweaty afterwards. They also stated that participating in uniform was not ideal. Wearing a uniform meant they felt hot and sweaty for the rest of the day or potentially being late to class if they changed clothing.

*“Yeah, it was boiling.“* (Female participant 1, Group 2)

*“… you would end up sweating for the rest of the day.”* (Female participant 3, Group 1)

[Would prefer] *“Not do it in uniform, because that was really annoying me … I had to quickly get changed.”* (Female participant 4, Group 1)

##### Moving beyond repetition

Regarding the specific activities, peer-recipients particularly enjoyed the boxing sessions but would have preferred more variety:

*“It was good – I liked* (when) *we did boxing.”* (Male participant 1, Group 2).

*“I think it should be like, a variety. Obviously, I do like boxing but I would rather have done like boxing on a Tuesday and then football or something on a Thursday.”* (Male participant 2, Group 2)

*“…instead of doing like a week of star jumps and then a week of doing, like, the boxing…You could have switched it over each day that we were doing it.”* (Female participant 3, Group 1)

##### Navigating exercise intensity through peer support

Overall, the peer-recipients liked and respected the Young Fitness Leaders, and acknowledged and appreciated the encouragement and understanding the Young Fitness Leaders had shown:

*“I liked the* [Young Fitness Leader] *that did my sessions, I like her…I liked* [Young Fitness Leader]* because she felt my vibe…She was relatable, like if I said I was knackered.”* (Female participant 3, Group 1)

(Liked a Young Fitness Leader) *“Because she’s encouraging, and I’ve known her for a while as well.”* (Female participant 5, Group 1)

“[Young Fitness Leader]* was just cool.”* (Male participant 2, Group 1)

*“Well, it was like, intense and he pushed you. Like he was like telling you ‘Come on. Come on, come on!’.”* (Male participant 2, Group 2)

#### Young Fitness Leaders' perspectives

Four themes were identified regarding the Young Fitness Leaders’ experiences. “Navigating session preparation and delivery” describes reflections on the pre-intervention training and how both the training materials and planning ahead of a session impacted session delivery. “Independence with a safety net” describes the tension between Young Fitness Leaders’ desire for independence while needing the security of adult (teacher/researcher) supervision. “Cultivating relationships” describes how the Young Fitness Leaders aimed to build rapport with the peer-recipients and the importance of this relationship. Lastly, “Looking to the future” describes Young Fitness Leaders' suggestions for future interventions.

##### Navigating session preparation and delivery

The Young Fitness Leaders found the pre-intervention training useful for preparing them to run HIIT sessions and felt the physical manuals were more useful than the online materials:

[The pre-intervention training] *“…definitely was useful. It wasn’t just, like, the activities, like demonstrating the different activities. It was, you know,* [dealing with] *the misbehaving individuals, the examples of that, that really helped.”* (Female peer-leader)

*“For me, my preference was using the manual that we were given…, I just believe that the manual it’s something easy that you can bring around with you, it’s easily accessible that you don’t worry about not being able to log on if you don’t have any wifi connection or anything like that.”* (Female peer-leader)

*“The book obviously had a lot of exercises, so just referring to that was really easy.”* (Male peer-leader)

Young Fitness Leaders appeared to prepare and plan for HIIT sessions in different ways:

* “…I don't think it* [session planning] *was too difficult. Like…you didn't have to go into depth thinking about it. So, you didn't have* [to] *sit yourself down and think about it for half an hour before you did the session. So, you could, you could, wake up before you went to school. You could just think in your head. Well, for me… I can't speak for anyone else, but like for me, I, I can just, I can imagine what I was going to do before I was doing it. Yeah.”* (Male peer-leader)

*“There was a few times where I’d come in on a morning, and obviously I’d already got my plan set out, but I was reading through the manual to double-check the times, to look at the different exercises that I could do if the kids didn’t like particular ones.”* (Female peer-leader)

When reflecting on the delivery of the sessions, all Young Fitness Leaders were able to articulate when they thought things had gone well:

*“This week both sessions were fantastic. They [*peer-recipients*] all put in a lot of effort and listened to everyone throughout the entirety of each session. The change in activity and exercise made a significant difference in their effort and behaviour so I believe boxing is a great activity to continue with.”* (Female peer-leader; Written Reflection, week 4).

*“They just seemed it* [excited]. *It was their body language and how they were in the sessions that* [they] *willingly took part. It seems like they were wanting to be there. Excited to be there in the morning.”* (Male peer-leader)

*“You knew what worked and what didn't and what was effective. So you know, if something's effective, like, what they'll enjoy, like doing sprints from side to side. You see they enjoyed that and then it works. So, you know, I'm going to start with that, so I'm going to finish with that because it gets, the pulse is raised* [a] *little, it looks good on the heart rate thing in the end.”* (Male peer-leader)

Fewer acknowledged instances where sessions had not run as well, however reflections centred around a need to more thoroughly plan sessions beforehand:

*“…some of them* [the sessions] *may not have gone as smoothly as we hoped, but that’s because us as Young Leaders weren’t as organised as we should’ve been”.* (Female peer-leader)

*“The session on Tuesday didn’t turn out the best, however that’s because the Young Leaders were unorganised. The session on Thursday went much better after improved preparation. Being given roles within the team (e.g. clock watcher, encouragement role, warmup role) helped us work more efficiently including the kids.”* (Female peer-leader; Written reflection, week 6).

One Young Fitness Leader identified that poor behaviour from a small number of peer-recipients was one of their concerns, although this concern was not widely reported:

*“There was* [sic] *two lads, just like faking injuries and just kind, they were still doing it, but trying to motivate them to run, which they would do in the end. But it was just difficult”.* (Male peer-leader)

##### Independence with a safety net

Encouragingly, the Young Fitness Leaders acknowledged the responsibility and independence of their role led to improved confidence in leading and speaking in front of others:

*“I definitely gained more confidence leading the group”.* (Male peer-leader)

[Gained]*“…communication skills with having to, like, engage them and tell them what with verbal communication and obviously visual as well to try and like demonstrate the activities”.* (Female peer-leader)

*“…there’s still that bit of pressure because you’re teaching, you’re teaching younger ones, you’ve got to be committed to doing so, but I actually found it really fun. It was nice to see everyone getting active, enjoying themselves.”* (Female peer-leader)

*“This made us feel a lot more responsible and independent and it definitely helped us in terms of gaining confidence in talking to them, and finding a way around difficulties when they don’t want to take part”.* (Female peer-leader)

Despite enjoying having the autonomy to run the sessions themselves, the Young Fitness Leaders also appreciated the presence of the researchers and teachers:

*“It was definitely something that we needed to be able to do by ourselves, but then knowing that yous* [sic] *are there for support as well it was definitely very helpful.”* (Female peer-leader)

*“I think I quite preferred the independence I got from teachers. Cause I think, if they were supporting us* [a] *bit too much, I feel like I just wouldn't have learned as much how to handle a group of Year 7s.”* (Male peer-leader)

##### Cultivating relationships

Young Fitness Leaders made a concerted effort to build rapport with the peer-recipients and made suggestions to improve engagement by getting peer-recipients’ input after the sessions. The peer-peer relationship was viewed by some as an important aspect of the success of the programme.

*“I just, tried to relate to them a bit more. You know, it was early in the morning, and they didn't necessarily want to be here, but I just, you know, I tried to relate to them and do the best I could.”* (Female peer-leader)

*“…giving them your trust and respect and they'll give it straight back to you and like will repay you with, like, hard work.”* (Male peer-leader)

*“I think we should probably talk to the kids more after the sessions and if they aren't enjoying it then, cause we we're never really properly spoke to them and asked them properly if they actually, what, what they think like are they enjoying it, are they actually getting anything from it.”* (Female peer-leader)

##### Looking to the future

The Young Fitness Leaders gave a range of suggestions for modifications that could be made to the intervention. Unlike the peer-recipients, the Young Fitness Leaders viewed the early morning scheduling of sessions positively:

*“I think it was better doing it in the morning because I think it fit into the schedule perfectly. I think* [with] *the lessons coming up and everything. I think it would've like prepared the Year 7 s more for school.”* (Male peer-leader)

Like the peer-recipients, the Young Fitness Leaders identified that adding some variety in activities may help engagement.

*“But I feel that if there were just a few extra activities that maybe could’ve involved equipment, like maybe skipping… if we could’ve used that … I know it’s a HIIT session… but if we could’ve added in extra things for them to do, and if they see equipment they’re like ‘oh my god new things, we can do new things!’ I feel like they would’ve enjoyed it a bit more. Not to say that they haven’t enjoyed it because I feel that they did, but…”* (Female peer-leader)

*“Most participants still choose to do boxing as their activity, but two girls wanted to go back to the different exercises. I took them to the side whilst the others were completing the boxing exercises and we did exercises such as high knees, jogging on the sport, squat jumps and heel flicks instead. This worked well and they contributed which was a positive”* (Female peer-leader; Written reflection, Week 6).

#### Teacher’s perspectives

Three themes were identified regarding the teacher’s perspectives. “Corridor Connections” describes the peer-peer relationships that formed amongst participants. “Hidden benefits of leadership” describes the perceived benefits of the programme for the Young Fitness Leaders. Finally, the theme “High interest low impact” describes the importance of maintaining student engagement while minimising disruption to the school timetable.

##### Corridor connections

The co-ordinating PE teacher (CT) and form teacher (FT) observed the development of positive relationships amongst the Young Fitness Leaders and between the Young Fitness Leaders and the peer-recipients:

*“It was really nice the collaboration that came across, like [*male peer-recipient] *was better when* [male Young Fitness Leader] was in…*and a couple of the girls were better when* [female Young Fitness Leader*] was in. It was the fact that they bult those relationships with the sixth formers, that was really good.”* (CT)

*They really respected the sixth formers…which I don’t think is an easy feat”. (FT)*

One teacher described that the formal programme led to informal positive social interactions throughout the school day:

*“We’ve spoken before about those relationships that were built between sixth formers and the Year 7s. I see them now, if* [Young Fitness Leader] *is walking down the corridor and he sees* [male peer-recipient]- *there will be a fist bump in the corridor…It promotes them looking out for each other. The young leaders will see them around and maybe it’ll give the young ones a bit of kudos as well, like- “Our young leader is a sixth former over there!” kind of thing. I think it’s made them more aware that other people exist when they are walking around in the school day.” (CT)*

##### Hidden benefits of leadership

The co-ordinating PE teacher noted potential benefits for the Young Fitness Leaders, including confidence, attendance and their own health:

*“I think its been really good for their confidence. And it got them in on the mornings sometimes where they might not have been.” (CT)*

*“I actually think it’s probably done wonders for their mental health during the day. I know it’s hard to measure that, like they do the surveys and things, I think that it’s those, it’s the hidden things that you can’t measure in numbers and figures. They are the key outcomes of this. It’ll inspire them to want to do it again, to want to do more of it.” (CT)*

*“It gave the sixth formers something to do to occupy their time which wasn’t just sitting in tutor time as well, like it was them active, them thinking, developing their “softer” skills and leadership skills. All round, the idea of it is great, it really is” (CT)*

##### High interest low impact

The teachers reported that a key strength of the programme was that it generated student interest, whilst maintaining a low logistical impact. The teachers perceived that the peer-recipients enjoyed the programme and that Young Fitness Leaders participated without requiring staff intervention:

*“They really enjoyed it I think because it was short and it was really focused.” (FT)*

*“…they did it positively, without having to be nagged, and they turned up every week, twice a week.”* (FT)

They also noted that the programme generated some organic interest with other children, potentially demonstrating a natural demand for future scale up:

*“There were a lot of kids who saw the set up in the canteen and were like “when’s it my turn”, “do I get to do this”, “I wanna box in the morning”, so like, actually its generated a little bit of interest between others in the school, and hopefully for them to be able to do that in the future.” (CT)*

The teachers described that a key advantage of the programme was the ‘containment’ within tutor time. This avoided disruptions to the core timetable which bypassed some of the usual barriers to school-based interventions:

*“It didn't take students out of lessons, it didn’t impact on their wider school education because it was contained within form*(tutor) *time, and because the tutor group got into the routine so quickly” (FT)*

Despite this, the co-ordinating PE teacher did identify logistical issues pertaining to accommodating the research project rather than the programme itself:

*“There were a lot of logistical things going on on my side which made it tricky as a teacher, but that’s more to do with probably the research side of things on your end and having all the paperwork…” (CT)*

With respect to future implementation of the programme, both teachers felt the programme in its current form fitted within the school day and could potentially be scaled up:

*“I couldn’t see why not [it could be scaled up] because it's not impacting on the timetable at all.”* (FT)

The co-ordinating PE teacher suggested the Young Fitness Leaders should be able to lead some smaller sessions early in the programme, and was also interested in how the programme might affect behavioural endpoints:

*“If you don’t give them* [Young Fitness Leaders] *the opportunity for them to be at the front, then they don’t make the mistakes and they don’t see what will potentially go wrong. I think if they had, a few, maybe three sessions, with a smaller group of kids beforehand, would’ve been good for them to get a handle on it” (CT)*

*“Let’s say we might have a tutor group with a couple of kids that are particularly hard work, we can have a look at their data how that looks in terms of behaviour, whether they’ve had less warnings or detentions or whatever that might be” (CT)*

### Secondary outcomes

### Intervention fidelity

#### Researcher field notes

Three themes were developed from the lead researcher’s observations of the HIIT sessions. “Evolution of independence” describes how in early weeks Young Fitness Leaders where often underprepared for sessions leading to protocol deviations requiring researcher input. “Fragility of the peer-led model” describes the impact that Young Fitness Leader absence had on intervention fidelity. Lastly, “The novelty gap” describes how peer-recipients behaviour impacted on intervention fidelity and how novel activities such as boxing mitigated this impact in the early weeks of the intervention.

##### Evolution of independence

First, the researcher noted that Young Fitness Leaders were often unprepared for their sessions in the first three weeks of the intervention; and required a lot of input from the researcher with respect to the choice and timings of exercises as well as motivating the peer-recipients. In many cases, the lack of preparedness manifested as Young Fitness Leaders deviating from HIIT activities provided in the manual (e.g., including activities such as burpees, triceps dips and supine bicycle legs), as well as incorrect timings of HIIT repetitions. This was despite Young Fitness Leaders consulting with their manuals in advance of sessions, though it was observed that the time spent on this varied across leaders. After additional feedback and encouragement from the researcher, along with refresher training in boxing-based HIIT at the start of week four, the Young Fitness Leaders became more independent and took ownership over the sessions. As the Young Fitness Leaders became more prepared and independent, there were fewer deviations to the protocol.

##### Fragility of the peer-led model

Second, the researcher also noted Young Fitness Leaders were occasionally absent, either due to school absence or because they forgot to attend. These absences were not always communicated, meaning the researcher had to assist with the running of the session. Peer-recipients would also sometimes miss their designated session because they wanted to attend sessions by a particular Young Fitness Leader.

##### The novelty gap

Third, the researcher also noted behavioural issues with some peer-recipients not focusing, wanting to sit out, or not trying very hard. Peer-recipients were more motivated and engaged during boxing sessions. This may have been because of the novelty of the activity. Engagement tapered off in some peer-recipients towards the end of the programme. Lastly, the inclusion of music during HIIT sessions negatively impacted the peer-recipients' ability to hear the Young Fitness Leaders' instructions. The music was therefore turned off from week two with no objections.

#### Intervention heart rate data

**Fig. 2:**
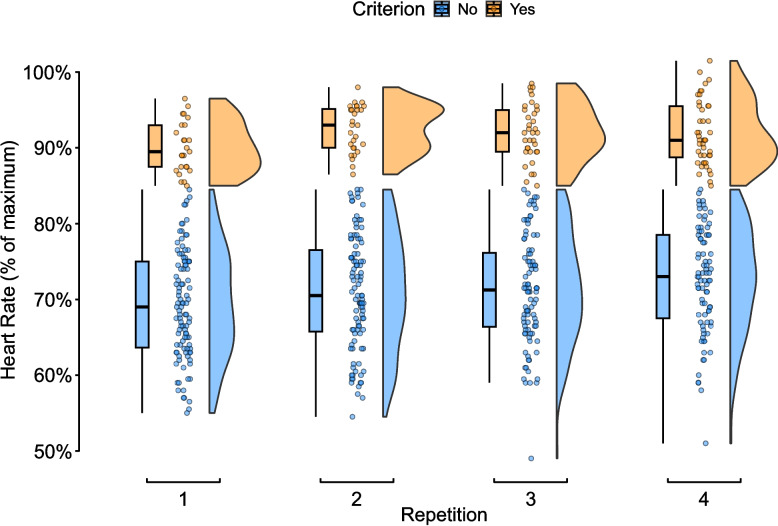
Raincloud plots of HIIT heart rate responses per repetition and categorised as either meeting (Yes) or not meeting (No) the HIIT heart rate criterion of 85% of age-predicted HR_max_

**Fig. 3:**
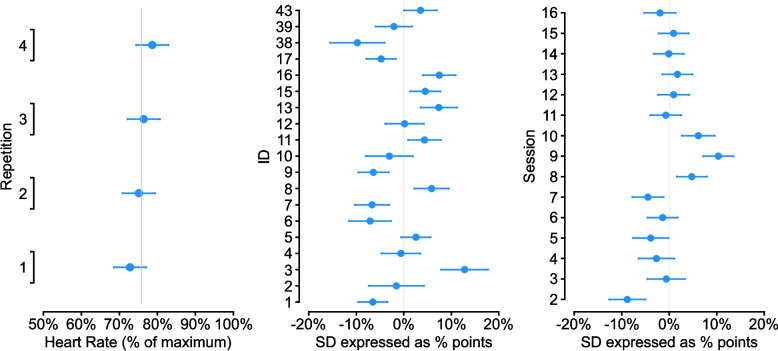
Fixed effect of repetition on heart rate, along with random effects, expressed as a SD, showing the variability in % points of age-predicted HR_max_ for participant and session. In all plots the grey vertical line represents the mean estimate

The proportion of intervention repetitions (*n* = 508) meeting our HIIT criterion was 26 (95%CI 22, 30) (bout 1: 19 [95% CI 14, 27]) %, bout 2: 22 [95%CI 16, 30] %, bout 3: 27 [95%CI 21, 36] %, bout 4: 35 [95%CI 27, 43] %) (Fig. 2). Mean repetition peak heart rate was 76 (95%CI 71, 80) % of age-predicted HR_max_, with trend for increasing heart rates with each subsequent repetition (Fig. 3). The between-participant SD was 6 (95%CI 4, 9) % points, 5 (95%CI 3, 7) % points for session, with a within-participant variability (residual) of 8 (95%CI 8, 9) % points. Figure 3 shows the model fixed and random effects, with the proportion of variance partitioned as 30% for participant, 17% for session, and 52% residual.

### Physical fitness and psychological outcomes

**Fig. 4:**
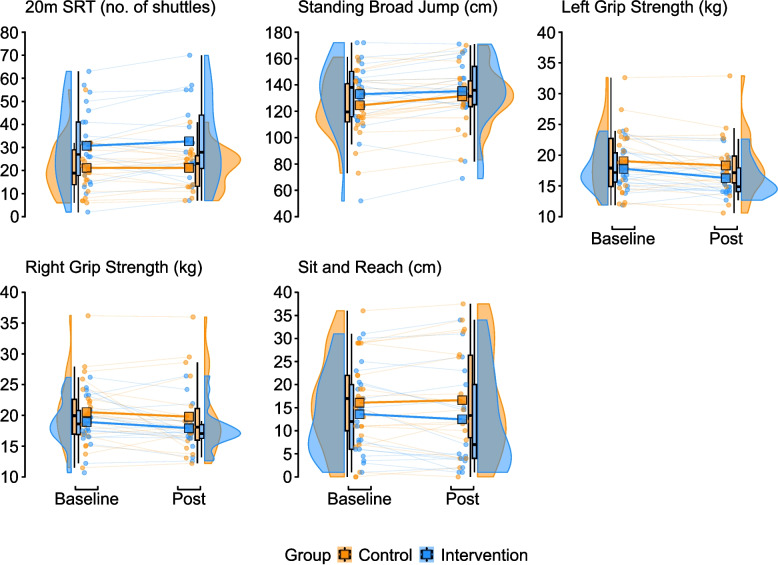
Raincloud plots of the baseline and post measurements for all physical fitness measures. In all plots, the mean group baseline and post values are shown via the appropriately coloured square

**Fig. 5:**
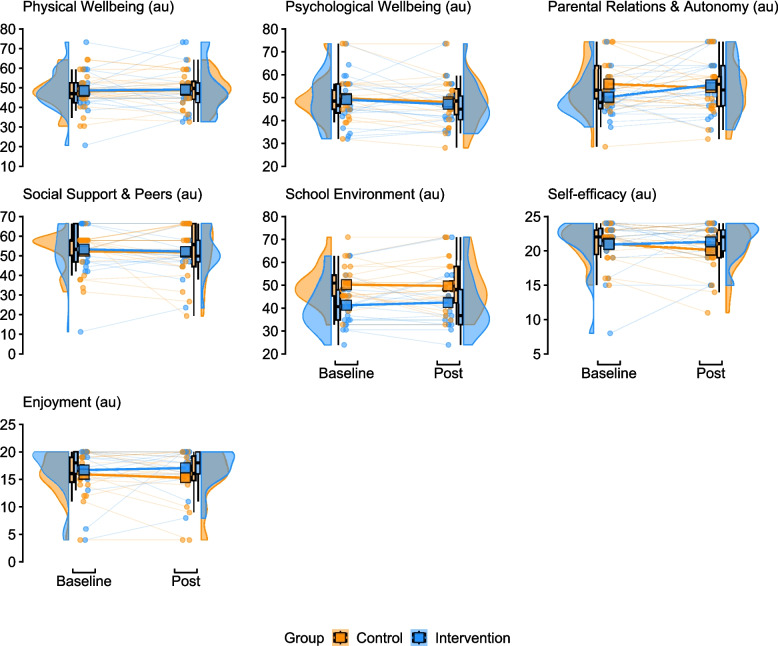
Raincloud plots of the baseline and post measurements for all psychological outcomes. In all plots, the mean group baseline and post values are shown via the appropriately coloured square. Arbitrary units = au

Baseline and post-intervention physical fitness outcome data for peer-recipients are presented in Fig. 4. The intervention effect on fitness measures was: 2.2 (95%CI −2.5, 6.8) shuttles for the 20mSRT performance, 0.3 (95%CI −2.0, 2.6) kg for right hand grip strength), −0.7 (95%CI −2.5, 1.2) kg for left hand grip strength, −0.2 (95%CI −2.7, 2.2) cm for sit and reach, and −1.9 (95%CI −9.0, 5.3) cm for the standing broad jump. Baseline and post-intervention psychological outcome data are presented in Fig. 5, and the intervention effect was: 0.9 (95%CI −5.3, 7.0) arbitrary units (au) for Physical Wellbeing, 0.7 (95%CI −4.9, 6.3) au for Psychological Wellbeing, 6.8 (95%CI −0.6, 14.1) au for Parental Relations & Autonomy, 2.7 (95%CI −4.2, 9.7) au for Social Support & Peers, 2.9 (95%CI −3.0, 8.9) au School Environment, 1.7 (95%CI −0.2, 3.6) au for Self-Efficacy, and 1.8 (95%CI –1.0, 4.7) au for Enjoyment.

## Discussion

The primary aim of our study was to explore the feasibility of a novel and potentially scalable peer-led HIIT intervention, where senior school pupils (i.e., Young Fitness Leaders) were trained to deliver HIIT sessions to younger pupils (i.e., peer-recipients) during morning tutor time. We also explored the fidelity of the intervention and the preliminary impact on physical fitness and psychological outcomes. Findings indicate the intervention was largely feasible from a recruitment, retention and attendance perspective; and well, though not universally, accepted by the peer-recipients, Young Fitness Leaders and teachers. Intervention heart rate data and session observations and reflections, however, suggest that intervention delivery and receipt could be improved before proceeding to a large-scale trial. For the physical fitness and psychological outcomes, the preliminary intervention effect directions were inconsistent and effect estimates imprecise. For the younger pupils (i.e., peer-recipients and control group participants), our recruitment rate of 73% is comparable to previous teacher-delivered HIIT interventions (e.g., [23, 24]) and other peer-led physical activity interventions conducted in a similar school climate and time to ours (e.g., [86]). Our retention of younger pupils was high (95%) and no intervention-related injuries or adverse events occurred. Our recruitment rate of Young Fitness Leaders was lower (42%; i.e., five of the twelve Year 12–13 pupils invited) and well below our target sample size of 10. While the recruitment of five and retention of four Young Fitness Leaders ensured the delivery of our intervention was still viable, lower recruitment could be attributed to several factors. Firstly, in contrast to other peer-led physical activity interventions (e.g., [86, 87]), our Young Fitness Leaders independently volunteered and were not pre-identified or chosen by school staff. To our knowledge, none held other school leadership roles, but two were youth football coaches and may therefore have felt better equipped to lead young people than those who did not volunteer. Indeed, our formative evaluation identified that some older pupils lacked experience and knowledge in how to lead and deliver exercise to younger pupils. While this aspect was included as part of the Young Fitness Leader’s training, initial recruitment may have been higher had the ongoing support peer-leaders would receive (i.e., before and during the intervention) been reinforced at the Year 12–13 recruitment session. To explore leader readiness and effectiveness using robust and objective measures, we recommend future studies administer questionnaires such as the Transformational Teaching Questionnaire [88], which was recently adapted for use in a primary school peer-led physical activity intervention [89]. Lastly, as we had intended to explore spillover effects of the intervention [89] on Young Fitness Leaders’ physical fitness and mental health, all were required to return a completed PARQ in addition to providing participant assent during the enrolment process. Though essential, this extra requirement may have hindered recruitment.

For peer-recipients, mean session attendance was relatively high (73%) and similar to previous school-based HIIT studies of a comparable intervention length [17]. In future studies, peer-recipients’ attendance and overall intervention experience could potentially be improved by scheduling HIIT sessions for later in the school day. This is reflective of peer-recipients’ perspectives that the early morning scheduling of HIIT sessions was the main negative aspect of our trial. In addition to concerns about being late and still tired at early morning sessions, peer-recipients did not like remaining in sweaty school uniform for the entire school day. The issue regarding school uniform impeding physical activity participation is long standing (e.g., [90, 91] and one we had hoped to avoid. At the intervention development stage, permission had been given for peer-recipients to wear PE clothing on scheduled HIIT days, which was reflective of Covid-19 mitigation strategies in place at the time. By the start of the intervention in mid-2023 however, these pandemic response measures had been removed, which necessitated peer-recipients wear their standard school uniform instead.

Despite dissatisfaction around the HIIT session timing, most peer-recipients were otherwise positive about their intervention experience. The enthusiasm displayed for the HIIT boxing activities mirrors positive participant feedback from a school-based HIIT study conducted in a similar geographical and sociodemographic area to ours [46]; and supports recent recommendations that school-based physical activity interventions are context-specific [92]. Peer-recipients did indicate they would like more variety in sessions if the intervention was to continue, which supports the researcher observations on the interplay of novel activities and continued engagement. This also reflects guidance on designing engaging school-based HIIT interventions [25] but was not possible to fully action in our trial, due to concerns about overburdening our small group of Young Fitness Leaders. In light of this feedback, and data from a recent study which reported improvements in adolescents’ motivation, enjoyment, affect and physical fitness components when choice and variety were provided for PE-based HIIT sessions [93], future studies should aim to provide recipients with greater autonomy in activity selection and variety.

Across the post-intervention focus groups, peer-recipients were overwhelmingly positive about their experiences and interactions with the Young Fitness Leaders, which sometimes extended beyond the HIIT sessions into the wider school environment. Review-level evidence suggests the attributes identified by the peer-recipients are important for the successful delivery of peer-led physical activity programmes (e.g. [29, 33, 34]). In the specific context of HIIT however, it is possible that exhibiting these behaviours during HIIT delivery could also help counteract some of the negative affective responses high-intensity exercise can elucidate [25, 46]. There is some preliminary evidence of this in our trial but requires further exploration. Encouragingly, reports of positive interactions extended into the post-intervention discussions with the Young Fitness Leaders and teachers. This was an unanticipated positive outcome of the trial and, given the recognised association between adolescent connectedness and mental health [94], is an important consideration for future studies. These interactions also align with aspects of social learning theory [95] and support the importance of relatable role models during adolescence [96]. Further, while our trial was not intended to be theoretically driven, to understand the peer-recipients’ engagement, findings from the peer-recipients could be interpreted through the lens of Self-Determination Theory (SDT) [97], which suggests that motivation is fostered when environments satisfy three basic psychological needs: autonomy (feeling a sense of choice), competence (feeling capable), and relatedness (feeling connected) [97]. Collectively, the findings from the peer-recipients indicate that while the Young Fitness Leaders were both autonomy- and relatedness-supportive for peer-recipients, environmental constraints (e.g. timing of the session and wearing uniform) were both autonomy- and competence-thwarting. This suggests that the quality of peer-recipients’ motivation and engagement could be impacted by the competing nature of the supportive social interactions versus the thwarting environmental factors [98]. Given this, future peer-led interventions could adopt a theory-driven design which may maximise engagement (of both peer-recipients and Young Fitness Leaders) while mitigating the impact of environmental constraints.

In contrast with the peer-recipients, the timing of the HIIT sessions was generally viewed favourably by the Young Fitness Leaders and teachers. For the Young Fitness Leaders this could potentially be explained by the differing expectations of their role compared to the peer-recipients, in that they were required to lead the HIIT sessions (e.g., demonstrate activities, provide encouragement and monitor timings), but not necessarily perform all the HIIT activities. For the teachers, the scheduling was attributed to improving morning school attendance for Young Fitness Leaders. While this observation was anecdotal and not formally quantified in our trial, they are key outcome considerations for future trials looking to maximise teacher- and school-management buy-in [99]. It was identified however, that scheduling sessions into such a short part of the school timetable meant there was little flexibility if sessions ran over. As this was mainly due to the time spent fitting and removing peer-recipients’ heart rate monitors at the start and end of the sessions, one potential solution is to provide peer-recipients with a wrist-worn physical activity and heart rate monitor after baseline testing, which they then wear for the entirety of the trial. This would negate the need to factor in time for monitor fitting and removal, yet still provide peer-recipients and Young Fitness Leaders with real-time heart rate feedback. Given recent advancements in analysing longitudinal data from device-based measures of physical activity [100] and the increasing use of wearables technologies in young people [101], this could also represent a viable alternative to hip-worn accelerometers for collecting continuous data on participants’ movement behaviours.

When exploring the Young Fitness Leaders’ intervention experiences, combining group insights (from the post-intervention focus groups), with individual reflections (from the weekly self-reflection task) enabled a more balanced and comprehensive account of how the intervention was delivered, and highlighted factors which likely impacted delivery fidelity [53]. For example, the group insights appeared to focus more on delivery aspects that went well. Comparatively, the individual reflections provided detail and reason for parts that did not go as intended, and often reinforced observations from the researcher that a lack of preparation negatively impacted delivery. As session planning and preparation was a fundamental component of the pre-intervention training, this suggests an element of social desirability bias in the Young Fitness Leaders’ group response [102]. Interestingly, group and individual insights were more aligned with regards to peer-recipients’ engagement and behaviour during the HIIT sessions. Here, both acknowledged that it was sometimes hard to engage certain peer-recipients who were putting in less effort or misbehaving, which was also identified as a key threat to fidelity in the researcher observations.

In addition to the researcher’s and Young Fitness Leaders’ insights on delivery, the peer recipients’ heart rate data enabled us to quantify the extent to which the HIIT sessions were performed as intended. As shown in Fig. 2, the proportion of repetitions which met our HIIT criterion of 85% of HR_max_ was 26%. Compared to other youth and adult HIIT studies which have adopted similar fidelity metrics to explore intervention receipt [35, 37, 47, 103], this is low and indicates the majority of repetitions did not meet our pre-specified HIIT criteria. The mean for peak heart rate across all repetitions was 76% of age-predicted HR_max_. While this is approaching the lower end of heart rate values associated with vigorous intensity physical activity (i.e., 77% of HR_max_; [104]), HIIT is typically performed towards the higher end of the vigorous intensity continuum. When compared to peak heart rate data reported in previous school-based HIIT studies utilising similar activities and protocols (e.g., [35, 47]) our average peak value was between 6% and 15% points lower. There is also evidence of considerable between- and within-participant variability in our data, suggesting the exercise dose was inconsistent between peer-recipients and across different points of the intervention.

While acknowledging the physiological limitations of using heart rate to quantify the intensity of short exercise repetitions [105, 106] and the caveat that our heart rate data represent only ~ 50% of the HIIT sessions, the HIIT session exercise intensity was insufficient. Aligning heart rate as a quantitative fidelity metric of how the intervention was performed, with the qualitative intervention delivery data, provides some useful insights as to why this occurred. Firstly, the Young Fitness Leaders sometimes deviated from the HIIT activites provided in the manual, instead including activities which were either not suitable for completing in school uniform (e.g. burpees), likely to be limited by local muscular fatigue (e.g., triceps dips), or unlikely to elicit a heart rate response indicative of high-intensity work (e.g., supine bicycle legs)—all of which could cause a decrease in exercise intensity. Deviations were most apparent when Young Fitness Leaders appeared under-prepared for sessions, which occurred more frequently in the early intervention weeks and before the boxing activities were introduced. As detailed in the researcher observations, this sometimes manifested as work:rest timings being incorrect, which could negatively impact the overall exercise dose, as well as intensity. Though we were keen to ensure Young Fitness Leaders had some autonomy in their session planning and activity selections, these deviations are examples of program drift [107] which can threaten the fidelity and effectiveness of an intervention [108], and has already been highlighted as a challenge to the successful scale-up of HIIT interventions for young people [25]. Further, while the Young Fitness Leaders attendance was high overall (mean attendance ± SD of 86 ± 16.4%), uncommunicated absences impacted the overall running of the HIIT sessions. This could lead to questions over the scalability of our current peer-led model, as the lead researcher was then required to assist with delivery. Finally, the Young Fitness Leaders seemed largely unaware of age-appropriate heart rate values that could be indicative of high-intensity work and the extent to which heart rate was expected to increase during the HIIT activities. As such, they could not maximise the usefulness of the heart rate monitors; both as an exercise intensity fidelity gauge and as a tool for providing real-time feedback and motivation to the peer-recipients [46, 109].

Collectively, we have highlighted some of the challenges of delivering a peer-led HIIT intervention with high fidelity across recipient and delivery agent metrics. Shortcomings in the majority of other school-based HIIT interventions, however, make comparisons in fidelity standards difficult. With a few notable exceptions (i.e., [23, 47]), school-based HIIT research has tended to quantify fidelity using training data only (e.g., heart rate, rating of perceived exertion and maximal aerobic speed) [17], thus focusing on how the intervention was received/performed, rather than how it was delivered. Given that most previous studies have been researcher or expert-led, this is perhaps unsurprising. If, however, a move towards scalable, non-expert led models is to continue, we strongly recommend that measures which examine intervention delivery, such as standardised fidelity checklists and/or observation checklists [47] are included alongside intervention performance data. While our trial fell short of using a standardised fidelity observations template, the in-depth researcher and Young Fitness Leaders’ insights could be used to tailor a peer-led HIIT-specific fidelity checklist for future studies.

Given the major role that fidelity can play in intervention effectiveness (e.g., [25, 80, 108], it is likely that the variability in our intervention effects reflect the varied delivery and receipt of the HIIT sessions. While the imprecision in our preliminary effects negate our ability to draw inference on the potential effectiveness of our trial, this was also not the primary purpose of our study. Nevertheless, the evaluation of a range of physical fitness and psychological outcomes alongside the feasibility aspects has provided useful insights which can inform decision-making for future trials. Though acknowledging our outcomes were not powered for inference, it could be that the inconsistent and imprecise changes observed for physical fitness outcomes were due to the HIIT sessions being performed at an intensity considerably lower than what we intended. This should be addressed in future trial iterations. Indeed, while review-level evidence indicates that improvements in cardiorespiratory and muscular fitness can be observed following school-based HIIT [16, 17], this is typically for studies with evidence of a consistently higher exercise intensity and/or overall exercise dose (e.g., longer intervals, greater number of sessions per week). Further, as our HIIT sessions were provided in additional to peer-recipients’ normal PE lessons and physical activities (as opposed to in replacement), a positive shift in physical fitness measures would not have been unreasonable. It is also possible that the intervention effects were confounded by movement behaviours performed outside of the HIIT sessions. Unfortunately, we cannot offer any insights on the likelihood of this, since poor accelerometer wear-time compliance left us unable to quantify the amount and intensity of physical activity the peer-recipients or control participants performed at any point during the trial. As both groups performed the same PE classes throughout the intervention period, it is less likely that this factor contributed, but we cannot comment on the role of physical activity behaviours performed outside of PE.

For the psychological outcomes, the intervention effect directions were more consistent, but effect estimates imprecise. It is also important to highlight that these trends are preliminary only and not powered for inference. Given concerns about young people’s mental health since the Covid-19 pandemic [2], and on-going debates about the perceived detrimental impact HIIT may have on psychological outcomes such as affect, enjoyment and future exercise intentions [25, 110] however, it is encouraging that our, albeit preliminary, data appear positive and does not appear to reflect the latter concerns. While further work is needed to refute or confirm these preliminary trends, it is possible they are reflective of the delivery methods employed by the Young Fitness Leaders during the HIIT sessions. Specifically, the positive feedback about the Young Fitness Leaders’ interactions and support of the peer-recipients may indicate that the sessions were delivered in line with the SAAFE teaching principles [45], which was a core component of the pre-intervention training. To explore this further, future studies could include behaviours reflective of the SAAFE principles within their session delivery checklists.

From the cautious interpretations of our secondary outcome data, and the in-depth insights gained on intervention delivery and receipt, we recommend that an enhanced iteration of our peer-led HIIT model is tested again, before deciding whether to proceed to a large-scale trial. By addressing the delivery and receipt issues at an early stage in the potential ‘scaling-up’ pathway, we may decrease the likelihood of observing a ‘voltage drop’ in outcome effects in a larger study, which has previously been reported as school-based interventions progress from efficacy to effectiveness to implementation [25, 111]. As intervention iterations can be context- and/or outcome-specific, we recommend four key modification areas for consideration. First, the peer-leaders’ training and support package should be enhanced, both pre-intervention and throughout the intervention period. On reflection, providing only one relatively short in-person training session for our Young Fitness Leaders was inadequate, given the delivery issues we encountered. While our timeframe was dictated by the school timetable, future peer-leaders would benefit from additional in-person sessions. As our trial may have positively impacted Young Fitness Leaders’ confidence, public speaking and leadership skills, future iterations could focus on developing effective leadership [112] before moving onto specialised exercise delivery, given the numerous benefits this important life skill is associated with [113]. Where possible, this could align with pre-existing school qualifications and/or opportunities in leadership acquisition, to maximise scalability and sustainability. For guidance on the delivery of such programmes, we direct readers to the recent ‘Learning to Lead’ intervention [89, 114]. While this was not HIIT-specific, improvements in peer-leaders’ leadership effectiveness, wellbeing, and time spent on-task in the classroom were reported alongside positive changes in peer-recipients perceived motor competence, school-based physical activity, and cardiorespiratory fitness [89].

Our second modification relates more specifically to the HIIT delivery training package. Given the Young Fitness Leaders’ positive feedback on the usefulness of our hard copy training manual, and researcher observations that the manual was often (if not always correctly) utilised in the early weeks of the intervention, we recommend this component remains a fundamental part of future training. Considering the lack of engagement with our supplementary online training videos, we do not view these as crucial components. Future studies may instead consider supplementing in-person training with smartphone applications, as were successfully utilised in the teacher-delivered HIIT interventions in Australia and New Zealand [23, 24]. As a means of including experiential learning activities [115] and providing peer-leaders with a better understanding of the physical cues associated with HIIT (i.e., heart rate values, bodily sensations), performing a full HIIT session while wearing heart rate monitors appears key [46, 109]. Here it might also be useful to include activities unlikely to evoke a heart rate response indicative of HIIT, to allow peer-leaders to see and understand why these are not appropriate, rather than simply being told not to do them. We also suggest that future programme extend the length of the HIIT session warm-up [116]. To enhance peer-leaders preparedness for HIIT sessions, it could also be helpful to run familiarisation sessions for peer-recipients and leaders before the intervention start date, akin to a trial run-in scenario [37]. This would facilitate opportunities for peer-leaders to practice their session planning and delivery and allow time for constructive feedback from researchers/teachers to be reflected and acted upon before the actual intervention.

The third modification attempts to address the behavioural issues we encountered, which may have impacted the fidelity of both the intervention delivery and receipt. While our issues are not uncommon and have been reported previously [46], it is recognised that unruly and overly demanding participants can disrupt group sessions, which can lead to a lack of participation by others [117]. For future studies therefore, we strongly recommend a teacher is present at all sessions and are therefore able to intervene with any behavioural/engagement issues in a timely manner. This would allow the beneficial social interactions between recipients and leaders to continue with minimal interruption. It could also facilitate peer-leaders’ autonomy in delivering the sessions independently with high fidelity, without distractions. Another potential mitigation strategy for behavioural issues is the addition of a PE teacher-led HIIT session alongside the peer-led sessions. This could allow for better behavioural modelling, since the PE-based sessions would take place in a more formalised learning setting. Further, as PE teachers will have more specialised knowledge and expertise in exercise delivery and working with young people, this addition could also lead to recipients receiving and performing activities at an intensity more reflective of high-intensity work. Since exercise intensity is often the essential component of an exercise intervention [80], this is an important consideration for interventions aiming to maximise changes in physical fitness outcomes. Careful consideration would have to be given to how additional and/or adjunct sessions are operationalised however, with a focus on keeping teacher and administrative burden low. Indeed, it is debatable whether a teacher-led session would have been palatable in our trial, since one of the key teacher positives was that the intervention did not impact on the wider school timetable.

Our final modification focuses on mitigating environmental constraints that could have negatively impacted the peer recipients’ autonomy and competence during the HIIT sessions, which were reflected in the lower than intended intervention heart rate data, and negative feedback about session timing and school uniform requirements. Where possible, we strongly recommend that sessions take place at a time point later in the school day. This could help address concerns over early morning tiredness, which in turn could lead to better engagement with the exercise protocol from an intensity, competence and enjoyment perspective. Considering the barriers to physical activity participation and engagement that school uniform creates, we also suggest that future studies either schedule sessions on days/times when participants are already wearing or have access to their PE clothing, minimise the time participants remain in clothes they exercise in, or allow for changing time within the session schedule. This could also lead to better engagement with the exercise protocol, as students may be less inclined to avoid bodily sensations associated with HIIT (i.e., sweating) if it is known they can change afterwards. Finally, to facilitate more variety and greater autonomy in exercise choice for peer-recipients without greatly increasing peer-leader workload, study recruitment efforts should focus on engaging a larger pool of peer-leaders with diverse activity interests.

In exploring the feasibility of a school-based peer-led HIIT model for the first time, our trial has highlighted some key considerations for researchers and practitioners looking to engage with young people as intervention delivery agents. We have also recommended four key modification areas for future iterations, based on the experiences of those who received, delivered and accommodated the trial within their school setting. Collectively, these are some of the key strengths of our novel and rigorously designed trial. Our study, however, is not without limitations. Due to the small number of Young Fitness Leaders, we are unable to comment on the impact of delivering the intervention on their physical fitness and psychological health. This has only been explored by a few others to date [28, 89] and is worthy of further examination. We also have no information on those who chose not to participate, so cannot comment on how to improve recruitment for future trials beyond the suggestions already provided. In adopting a non-randomised study design, we are aware that selection bias (where systematic differences in the treatment groups arise at baseline) might have occurred. This threat to validity was at least partly assuaged by adjusting for baseline imbalance in the statistical analysis [83]. Given this was a feasibility study with a relatively low sample size, we recommend all intervention effects are interpreted with caution. Nonetheless, following our suggested iterations to the intervention model, we recommend that these outcomes are further examined in future studies. As our trial was conducted in one secondary school located in a relatively deprived area of North East England, we acknowledge that the generalisability of our findings to other areas and settings may be limited.

## Conclusions

From an overall recruitment, retention, attendance and acceptability perspective, our findings indicate that peer-led HIIT may represent a scalable and feasible school-based physical activity model. By working with senior school pupils as intervention delivery agents, our trial provides a novel and viable model for school-based HIIT scale-up, without substantially increasing teacher burden. The intervention contents were largely well received by peer-recipients and there was evidence of positive interactions between themselves and the Young Fitness Leaders extending beyond just the HIIT sessions. Given the negative feedback related to the timing of the intervention, we suggest this is adjusted in future trials. The Young Fitness Leaders spoke positively about their experiences of delivering the HIIT sessions, but intervention fidelity metrics suggest future peer-leaders would benefit from further support in preparing for intervention delivery and eliciting heart rate responses indicative of high-intensity work. This highlights the complexities associated in designing an intervention with potential for scale-up and should be addressed in future iterations of our peer-led HIIT model before progressing to a large-scale trial.

## Supplementary Information

Supplementary Material 1.

Supplementary Material 2.

Supplementary Material 3.

Supplementary Material 4.

Supplementary Material 5.

Supplementary Material 6.

## Data Availability

The datasets generated during and/or analysed during the current study are stored in a publicly available repository (https://data.ncl.ac.uk) and will be made available once the study finding have been published. Qualitative datasets are redacted where necessary to protect participants' anonymity.

## Abbreviations

20 m SRT: 20 M Shuttle Run Test
95% CI: 95% Confidence Intervals
ANCOVA: Analysis of Covariance
au: Arbitrary Units
BMI: Body Mass Index
CERT: Consensus on Exercise Reporting Template
CT: Coordinating teacher
FT: Form Teacher
HIIT: High Intensity Interval Training
HR_max_: Maximal Heart Rate
HRQoL: Health-Related Quality of Life
PARQ: Physical Activity Readiness Questionnaire
PE: Physical Education
RPE: Rating of Perceived Exertion
SAAFE: Supportive, Active, Autonomous, Fair, Enjoyable
SD: Standard Deviation
TIDiER: Template for Intervention Description and Replication
UK: United Kingdom
YFIT: Youth Fitness International Test
