# Supplementary material for: Feasibility of a school-based peer-led high-intensity interval training intervention: the Young Fitness Leaders project
Source: BMC Public Health. 2026 Feb 24;26:799. doi: 10.1186/s12889-026-26543-w (PMC12961876; doi:10.1186/s12889-026-26543-w)
Supplement: Supplementary file 1 — Supplementary Material 1. [file 12889_2026_26543_MOESM1_ESM.docx]

**Pre-trial formative evaluation: Young Fitness Leaders project**

**Focus group facilitators:** Hannah Batten and Laura Basterfield

**Purpose:** To utilise the insights and ideas of school pupils and teachers to enhance and refine our preliminary intervention plans (Gittelsohn et al., 2006).

**Protocol and findings overview**

**Site:** One secondary school in Ashington, Northeast England which is one of the 10% most deprived locations in England (Noble et al., 2019).

**Participants:** Pupils who provided informed parental/guardian consent and individual assent.

**Number of focus groups and group member details:** Four focus groups were conducted. The first group consisted of Year 7 pupils (age 11-12 years, n=4; representative of the intended target population for peer-recipients), the second of Year 8 pupils (age 12-13, n=5; representative of the intended target population for peer-recipients) ) and the third of Year 9 and 10 pupils combined (age 13-15 years, n=8; representative of the intended target population for peer-recipients)). The fourth group consisted of pupils in Years 12-13 (aged 16 - 17 years; representative of the intended target population for peer-leaders).

**Focus group questions:**

Across all focus groups, participants discussed the qualities of a peer-leader, the suitability of Year 12-13 pupils to deliver a peer-led intervention, perceptions of HIIT, and ways in which HIIT could be performed at school. They also discussed intervention logistics. In the Year 12-13 group only, participants shared perceived barriers and facilitators to delivering a peer-led HIIT intervention and potential benefits and drawbacks to participation. For participants in the Years 7-10 groups, perceived barriers and facilitators were discussed in the context of receiving a peer-led HIIT intervention.

In addition, the physical education (PE) teacher assisting with the trial co-ordination took part in a semi-structured interview and 12 other school staff members completed a short, written survey with open text responses. In both instances, questions were broadly equivalent to those asked in the pupil focus groups, but through the lens of a teacher rather than a peer-recipient or peer-leader. Teachers were also asked if they foresaw any benefits or drawbacks to their pupils’ involvement.

**Data analysis**

The pupil focus groups and PE teacher interview were audio recorded and transcribed verbatim. Text responses from each survey question were merged into a word document. All data were analysed using inductive thematic analysis (Braun and Clarke, 2022) by one researcher (Naomi Burn), then checked by a second researcher (Kathryn Weston). Data from each distinct group (i.e., Year 7-10 pupils, Year 12-13 pupils, and teachers) were analysed separately. The key findings are summarised overleaf.

**Findings:**

**Potential peer-recipients**

When describing a peer-leader, potential peer-recipients (i.e., pupils in Year 7-10) listed qualities such as being trustworthy, funny, non-judgmental, relatable and having good communication skills. They thought Year 12-13 pupils could have these qualities and described their proximity in age as a facilitator to engaging in a peer-led programme. The main barriers identified were a mismatch in their exercise ability and peer-leader expectation/instruction, awkwardness in front of older pupils, and older pupils acting ‘superior’ to younger ones. The latter comment was made during the Year 9-10 focus group, this came from the Year 9-10 focus group and was said in the context of concerns that older pupil’s standards might be higher than younger ones. While it was apparent that the youngest participants (i.e., those in Year 7) were less familiar with the concept of HIIT than those in Years 8-10, the majority were willing to try it and suggested a variety of bodyweight exercises and sporting activities for incorporation into the intervention. With regards to intervention timing, it was generally thought that sessions should take place at some point before lunchtime, but a single most popular timeslot was not identified. Participants thought HIIT sessions should take place outside or in the school sports hall, depending on the weather. For exercise group size, the overall preference was for smaller groups, rather than the entire class.

**Potential peer-leaders**

These logistical aspects largely mirrored discussions in the Year 12-13 group where participants thought they had peer-leader qualities but would not feel comfortable leading large groups. These participants were familiar with the concept of HIIT but highlighted that exercises such as burpees and mountain climbers might not be suitable to complete in school uniform. Their main barriers to delivering HIIT were feeling underprepared, lacking knowledge and experience of working with young people and managing poor behaviour. They identified these barriers could partly be addressed through training opportunities and ensuring they were organised for sessions. They felt that delivering a peer-led intervention could improve their confidence, leadership skills and be useful for future university/ workplace applications.

**School teachers**

This view was shared by the teachers, who were generally very supportive of the proposed trial, but voiced some concerns about the Year 12-13 pupils’ timekeeping and school attendance. They also highlighted the importance of working with smaller numbers (i.e., classes from only one year group) in the first instance and suggested the school morning break time (1030-1100 hours) as a time when HIIT sessions could run.

**References**

Gittelsohn J, Steckler A, Johnson CC, Pratt C, Grieser M, Pickrel J, Stone EJ, Conway T, Coombs D, Staten LK: Formative Research in School and Community-Based Health Programs and Studies: 'State of the Art' and the TAAG Approach. *Health Education & Behavior* 2006, 33(1):25-39.

Noble S, McLennan D, Noble M, Plunkett E, Gutacker N, Silk M, Wright G: Ministry of housing, communities and local government. *The english indices of deprivation* 2019.

Braun V, Clarke V: Conceptual and design thinking for thematic analysis. *Qualitative psychology* 2022, 9(1):3.
