# Supplementary material for: Feasibility of a school-based peer-led high-intensity interval training intervention: the Young Fitness Leaders project
Source: BMC Public Health. 2026 Feb 24;26:799. doi: 10.1186/s12889-026-26543-w (PMC12961876; doi:10.1186/s12889-026-26543-w)
Supplement: Supplementary file 2 — Supplementary Material 2. [file 12889_2026_26543_MOESM2_ESM.pdf]

YOUNG FITNESS LEADERS MANUAL

2023

Edinburgh Napier
UNIVERSITY

Newcastle
University

THANK YOU FOR BEING A YOUNG LEADER

We appreciate your dedication and commitment to this project and with your help we are sure it will be a success.

WELCOME MESSAGE

Congratulations on being part of this exciting project!

You are set to make a significant impact on the health and well-being of your school community.

We hope you enjoy this journey and take pride in the important role you will play in promoting fitness and well-being at your school.

This guide will answer any questions you may have about being a Young Leader in this study and show you how to perform and lead the different exercises you'll need.

If you have any further questions please contact Laura Basterfield at Newcastle University: laura.basterfield@ncl.ac.uk

Copyright Newcastle University. No part of this manual may be copied, reproduced or distributed without permission from the authors.
With thanks to Professor David Lubans at the University of Newcastle, Australia, and the Burn2Learn programme.

TABLE OF CONTENTS

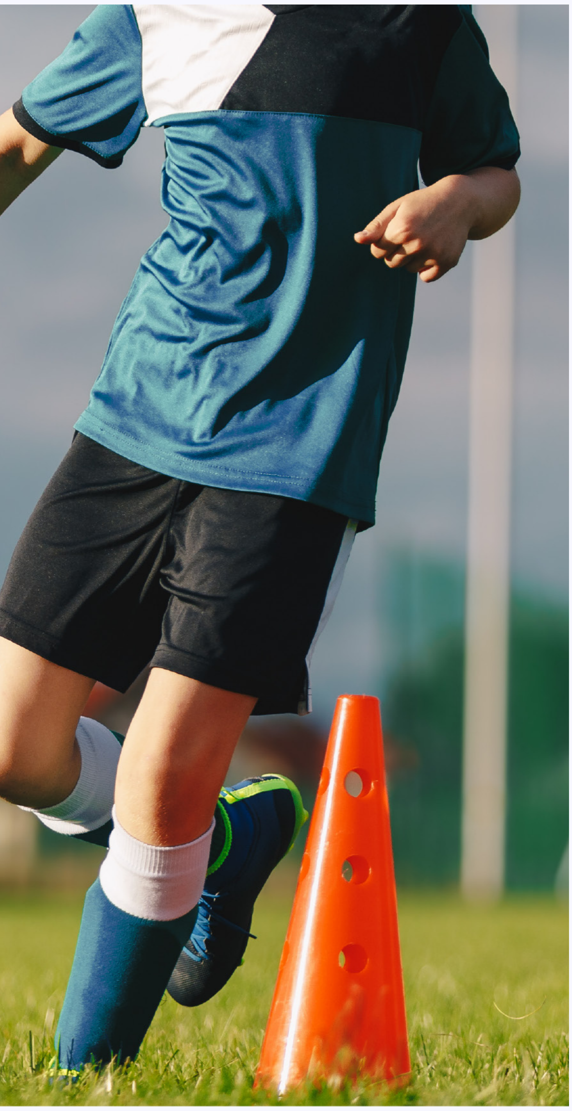

4	What is this project about?
4	What is high-intensity interval exercise training?
5	What will you do as a Young Leader?
6	Vision and mission
7	Support and professionalism
8-9	Structure of a session
10	Health and safety
11-31	HIIT ideas and how to demonstrate them
32	Things to consider when running your sessions
33	Reflections
34	Session plan

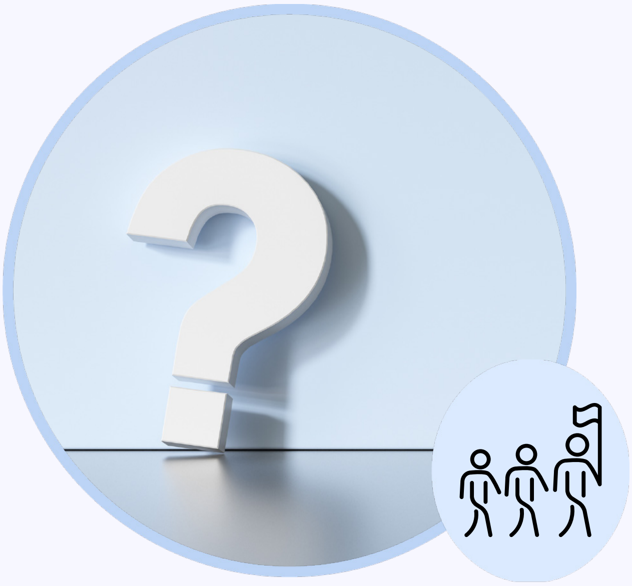

WHAT IS THIS PROJECT ABOUT?

This project aims to train Year 12 and 13 students to be ‘Young Fitness Leaders’. The Young Leaders will take short activity sessions, based on high-intensity interval exercise, for younger pupils, 2-3 times a week during morning tutor time. This project will last around 8 weeks, but we hope it is something that could continue at school all year round.

We spoke to lots of pupils to help us design the programme; we have included as many of their ideas and opinions as possible.

This is a ‘test run’ of the project, allowing the researchers to work out the practicalities, and assess whether it could work on a bigger scale.

WHAT IS HIGH-INTENSITY INTERVAL EXERCISE TRAINING?

The type of exercise you will lead in this project involves rounds of short bursts of intense exercises which raise your heart rate and make you breathe faster, followed by a recovery period.

This is known as high-intensity interval exercise training, or HIIT.

To be classed as HIIT, an activity needs to increase the heart rate to around 85% of maximum. In a Year 7 pupil this would be around 177 beats per minute.

HIIT can improve cardiorespiratory fitness, body composition and metabolic health, and HIIT programmes have been done in schools around the world.

WHAT WILL YOU DO AS A YOUNG LEADER?

You will need to commit to leading a HIIT session twice a week for a small group of 5-6 Year 7 students. The sessions will take place during morning tutor time (8.30am).

You will need to be in the location at the right time, as morning tutor time is short! The Year 7s will meet you there.

The sessions are described in more detail later, but briefly you will do:

- ## *WARM UP*

A 2-minute warm-up activity.

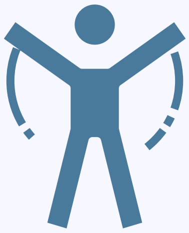

- ## *EXERCISE*

45 seconds of intense exercise

(Ask the year 7s to chose from the list in advance)

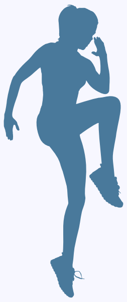

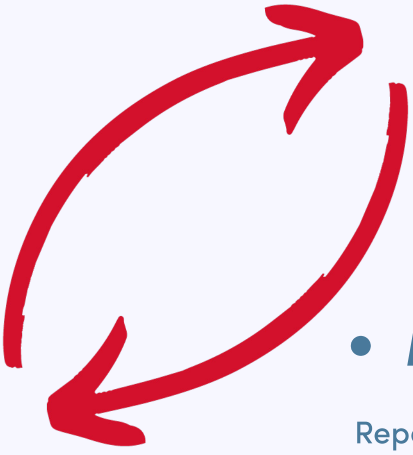

- ## *REST*

Rest for 60 seconds

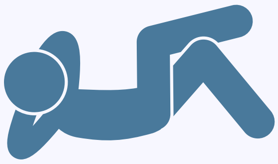

- ## *REPEAT*

Repeat the protocol 4 times

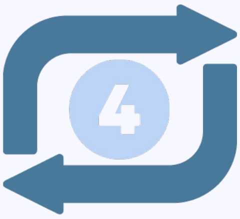

VISION & MISSION

As this is a trial to help us improve future versions of the project, we would like you to complete a brief reflection at the end of each week.

Think about how you felt the session went...

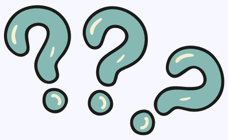

- What worked well?
- Did you face any challenges?

We will give you a short reflection template to complete.

Role models

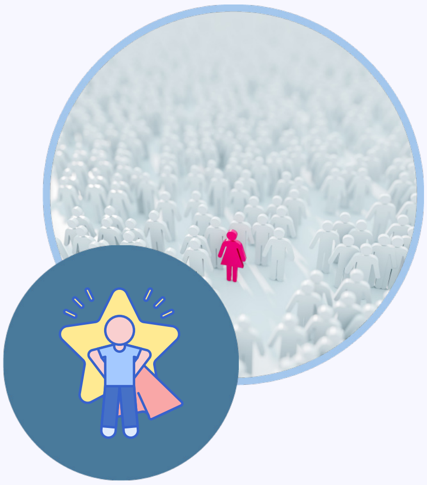

The Year 7s are looking to you for guidance, so it is important that you are **approachable** and **considerate**.

Plan your sessions by asking in advance which exercises they want to do, and which music they would like.

Ask after the sessions if there are any exercises they really don't like, or if you notice that they don't seem to be enjoying an exercise.

How to monitor exercise intensity

It is really important that the Year 7s are working at a high intensity to get the physical and mental benefits.

HIIT looks different in each of us, but Young Leaders can ensure the Year 7s are reaching the correct exercise intensity by looking for visual signs such as **getting out of breath**, **sweating**, and being **red in the face**.

Give lots of encouragement throughout each session to help the Year 7s give their best effort.

If anyone is too tired to complete a HIIT activity, they can rest early and recover ready for the next one.

SUPPORT

• WHAT SUPPORT WILL I BE GIVEN?

Researchers will be at each of your sessions, and you can contact us in between sessions – details are at the front of this guide.

You are **NOT** responsible for the behaviour or conduct of the Year 7s – you are there to lead a HIIT session.

If you have difficulties with the pupils in your group please let the research team or Mr Hunter know immediately.

If you experience difficulties with any of the exercises or timing equipment, let us know as soon as you can so we can fix them.

The researchers will check session attendance, and giving some Year 7s a heart rate monitor to wear on their wrist. They will also ask some of the group to report how tired they feel after a session using a picture scale.

• PROFESSIONALISM

As a Young Leader it is important that you behave in a professional, appropriate and respectful way towards the younger students.

BEFORE: be in the correct location before the Year 7s arrive, have timers or equipment ready to go!

DURING: monitor technique and effort, check times, be enthusiastic!

AFTER: pack everything away ready for next time, leave the area clear and safe again.

If you are unwell on the day, **please arrange for another Young Leader to cover your session.** If you are unable to do this, let Mr Hunter or the research team know as soon as possible.

STRUCTURE OF A SESSION

The first two weeks will look like this:

Time (minutes into tutor time)	Activity
0-1	Year 7s arrive.<br>Researchers mark register.
2-4	90 second – 2 minute warm-up and chance to practice some of the activities you'll do in the main HIIT session.<br><br>Choose some of the activities you'll use (e.g. high knees, lunges) and do one for around 15 seconds, then move on to the next (no rest) until you have completed between 90 and 120 seconds of activity. These should be completed at a slower pace than during the HIIT session and focus on warming up the body and practicing movements with good technique. Now move onto the main part of the session:
5	45 second HIIT activity
6	1 min rest
7	45 second HIIT activity
8	1 min rest
9	45 second HIIT activity
10	1 min rest
11	45 second HIIT activity
12	End!<br>Everyone have a drink of water and head off to class

STRUCTURE OF A SESSION

After 2 weeks we will increase the time of the activity by 5 seconds every two weeks, so that it looks like this:

Time (minutes into tutor time)	Activity
Weeks 3 and 4	50 second exercise burst
Weeks 5 and 6	55 second exercise bursts
Weeks 7 and 8	60 second exercise bursts

Make sure to enter the new times into your timer.

HEALTH AND SAFETY

Check your area:

- Is the floor dry?
- Are there trip hazards like bags or clothes?
- Is there enough space?

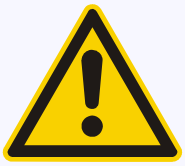

Encourage everyone to remember their water bottle, too.

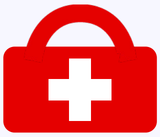

If **anyone feels unwell during the sessions,** they can **stop immediately and rest.** If they need help after the session, take them to the office. The research team will also be there.

As this is high-intensity exercise, it is possible that people may have sore legs the next day, **but this is normal and will wear off.** However, anyone experiencing **sharp shooting pains should stop immediately and one of the research team will come over and check them.**

HIIT IDEAS AND HOW TO DEMONSTRATE THEM

When selecting an exercise, it's really important to first consider:

- **Does your group enjoy it? Check with them beforehand.**
- **Can it be done in school uniform?**
- **Has the group requested their music?**

If you can answer 'yes' to all of these, get ready to move!

Edinburgh Napier UNIVERSITY

Newcastle University

JUMPING JACKS

1. Stand straight with arms at your side

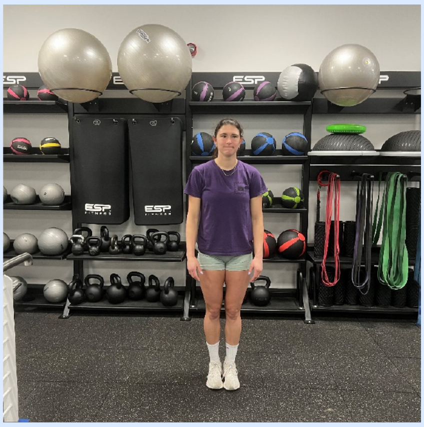

2. Perform a small jump upwards, while bringing your legs wider than your hips and your arms overhead.

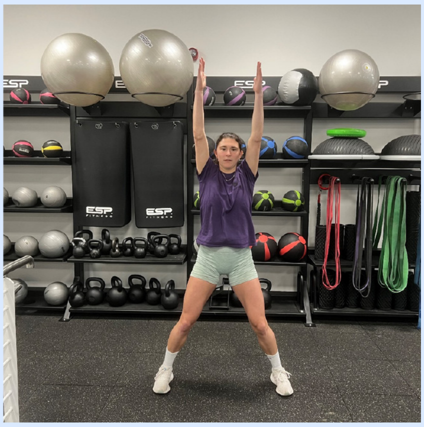

3. Jump again, returning your arms to your sides and your legs back to the starting position.

4. Continue this jumping outwards and inwards to perform a jumping jack.

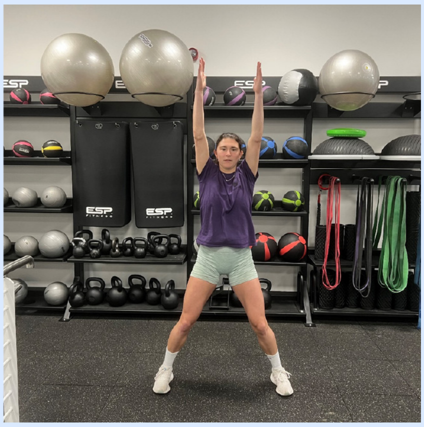

STAR JUMP

1. Start by performing a shallow squat

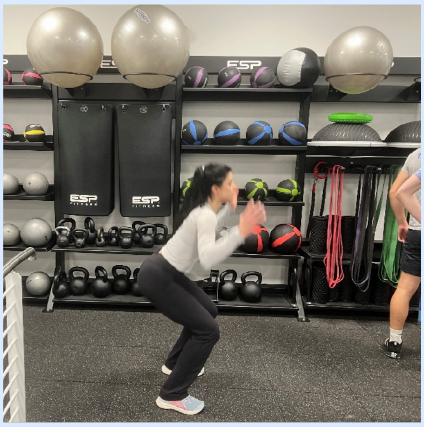

2. Jump as high as you can while extending arms and legs diagonally.

3. You will look like a star while in the air.

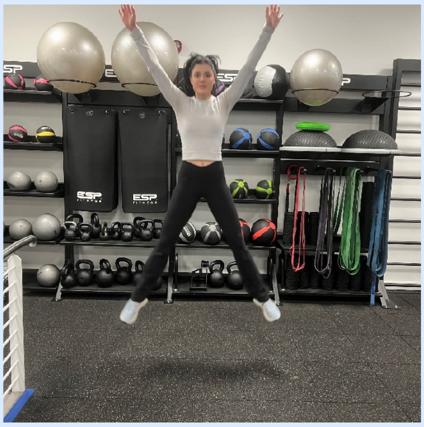

4. Bring limbs back towards your body to land, this will bring you back to the starting position.

Easier modification: jump lower.

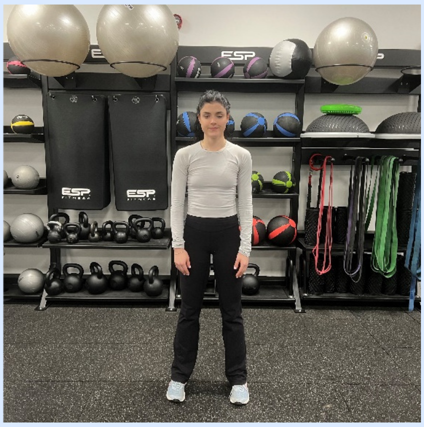

LUNGES

1. Stand with feet hip-width apart

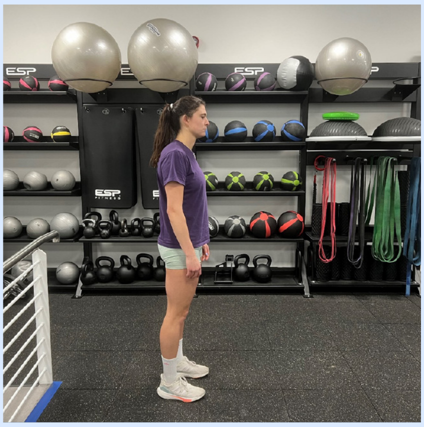

2. Take one big step forward

3. Hips and torso facing forward with one leg in front and one behind

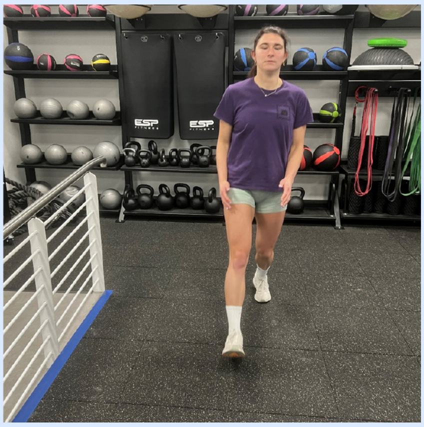

4. The back knee is bent at a 90 degree angle hovering over the ground

5. The front foot is firmly planted on the ground, with the knee at a 90 degree angle.

6. Push off the front leg to return to the starting position.

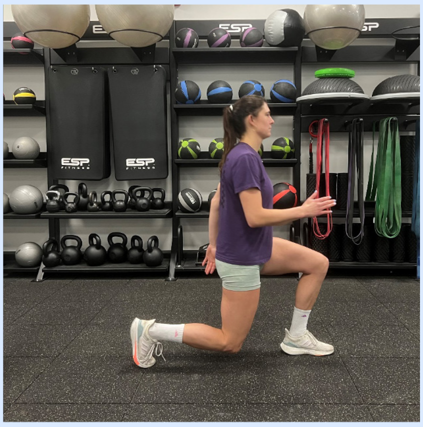

LUNGES -MODIFICATIONS

Easier modification

1. Take one big step forward
2. Hips and torso facing forward with one leg in front and one behind
3. Lower rear knee as far as you can comfortably
4. Front foot is planted and lowering as far as you can comfortably
5. Push off the front leg to return to the starting position

Harder modification

Modify to lunge jumps

Edinburgh Napier UNIVERSITY Newcastle University

SQUATS 1

1. Stand with your legs shoulder width apart

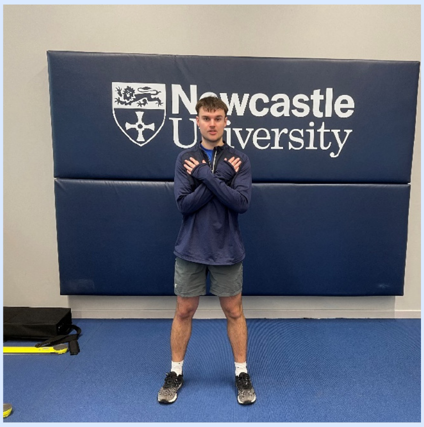

2. Begin to sit back as if you are sitting in an invisible chair, with your feet firmly on the ground

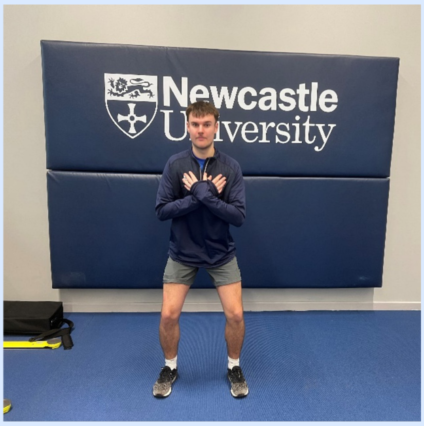

3. Sit back until your knees have hit a 90 degree angle

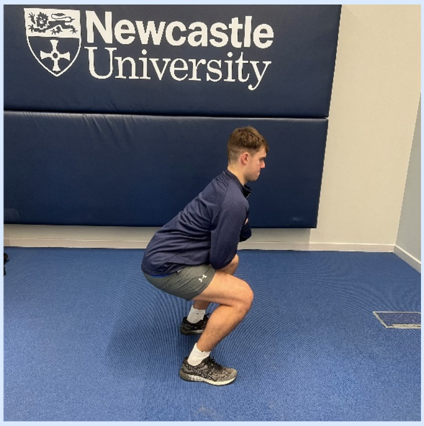

SQUATS 2

4. Press feet into the ground and ensure your knees don't come inwards when pushing out of the squat to return to the starting position.

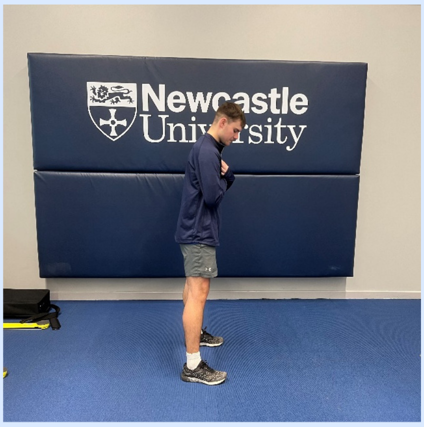

Edinburgh Napier UNIVERSITY

Newcastle University

SQUAT JUMPS 1

1. Perform a semi-squat with feet hip-width apart.

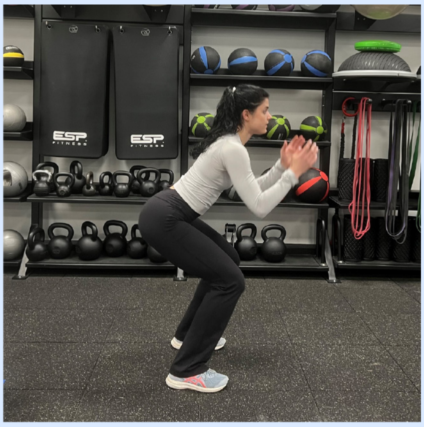

2. Arms can be at your side or in front of your body.

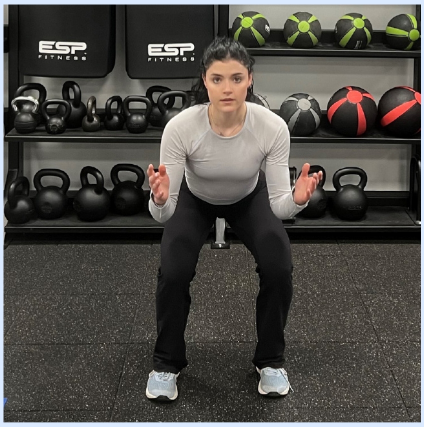

3. Explode out of the squat to jump as high as you can.

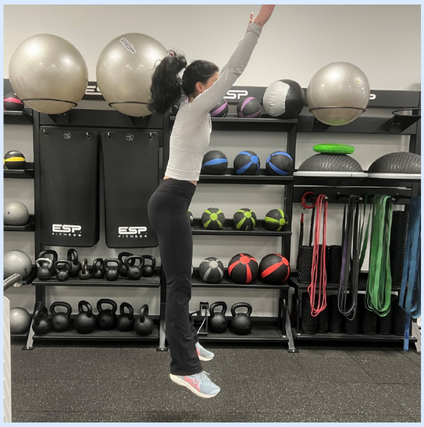

SQUAT JUMPS 2

4. Return to the semi-squat to make it continuous.

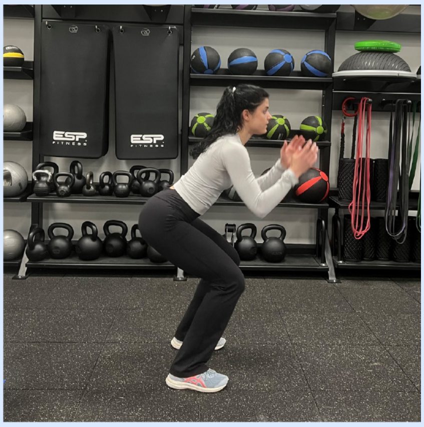

Easier modification

Perform a squat jump, but rather than making it continuous you can ‘reset’ after each rep.

Harder modification

Rather than semi-squatting, perform a squat to a 90 degree angle and a max explosive jump. Perform this continuously

HIGH KNEES

1. Drive your alternating knees towards your chest.
2. This can be compared to a fast paced high knee march.

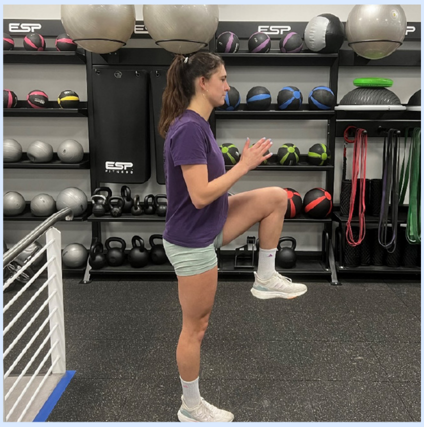

3. Drive arms forwards and backwards.

Can be performed in place or from a start line to a finish line.

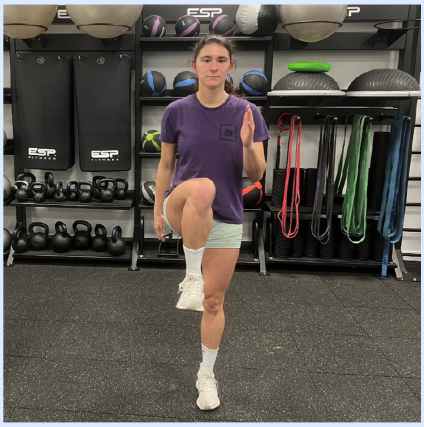

Easier modification:

Slow the pace of the high knees, but ensure you are driving your knee at least to a 90 degree angle.

Harder modification:

Increase the pace of your high knees. Driving your knee towards the sky every step. Try to get as many reps in as possible.

SKIPPING

1. Add a small hop in each step, driving each alternating knee up slightly.

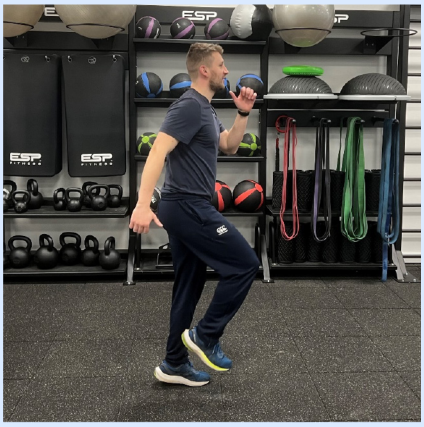

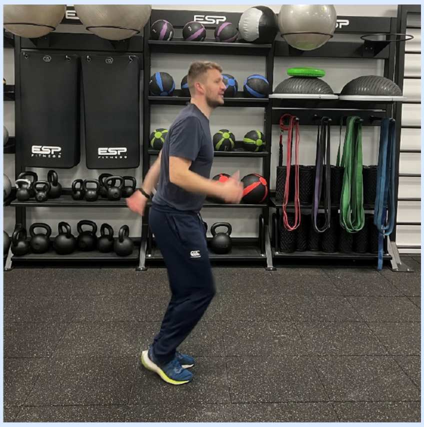

2. Alternate which knee is going up and which leg is performing the hop

3. Repeat this motion

Harder modification:

Increase the height of your hop

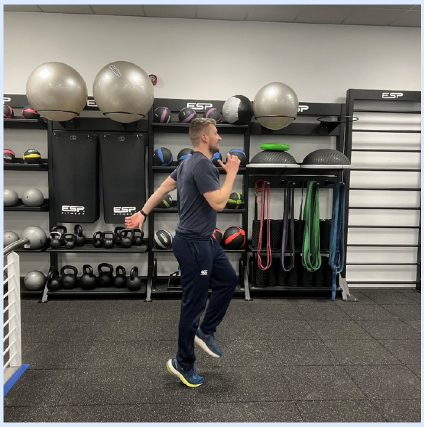

RUNNING ON THE SPOT

1. Find a spot where you will perform a run in place.

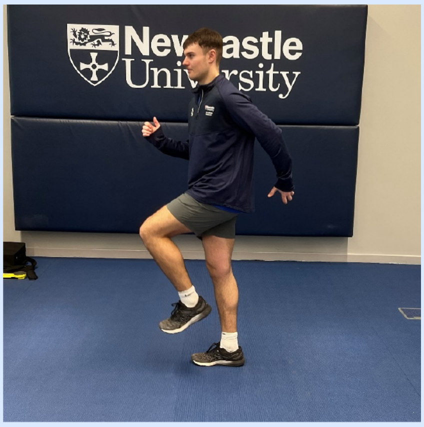

2. Alternate your arms and legs.

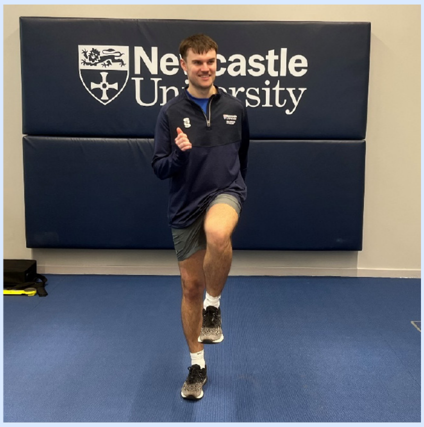

Harder modification:

Increase speed of your running

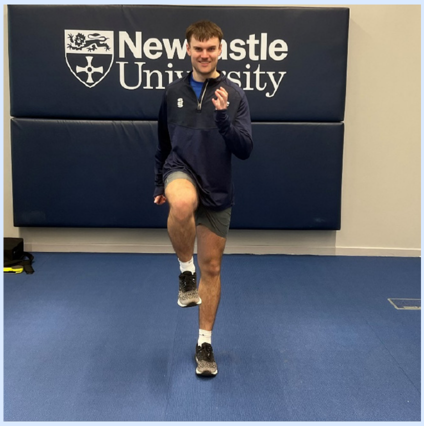

SPRINT

1. Perform a maximum effort run from a start line to a finish line

BOXING 1 - HAND POSITION

1. When preparing to box, make sure to keep your thumb wrapped OUTSIDE of your clenched fingers to avoid injury. Correct position shown in picture.

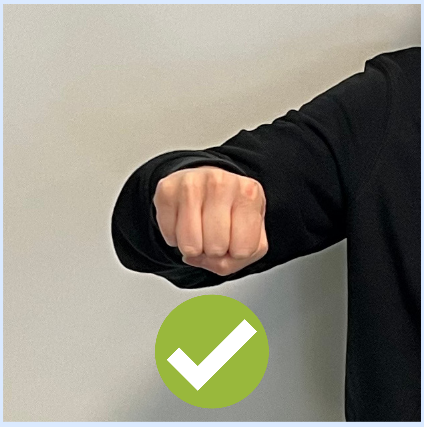

2. Incorrect position: thumb covered by clenched fingers

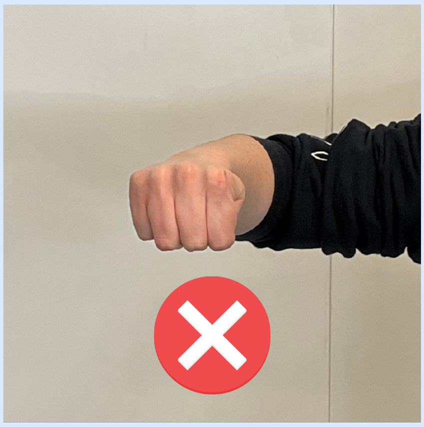

3. Make sure thumb remains OUTSIDE of clenched fingers once glove is on too.

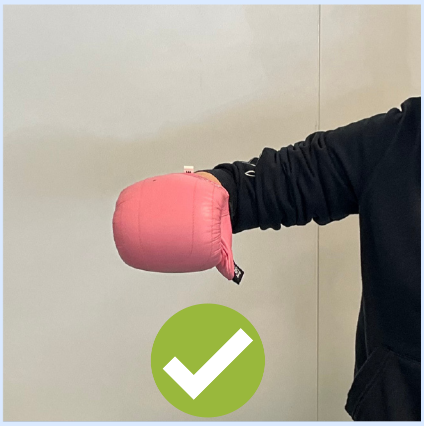

Edinburgh Napier UNIVERSITY

Newcastle University

BOXING 2 - HAND POSITION

Make sure thumb remains OUTSIDE of clenched fingers once glove is on too.

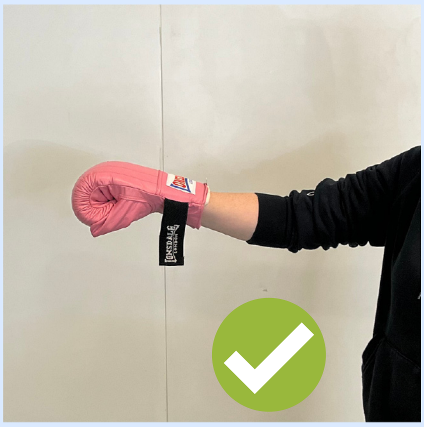

Incorrect position: thumb covered by clenched fingers

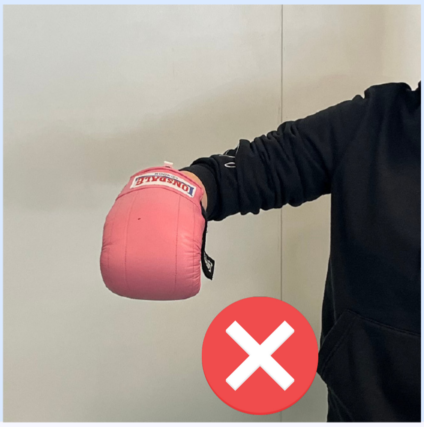

Incorrect position: thumb covered by clenched fingers

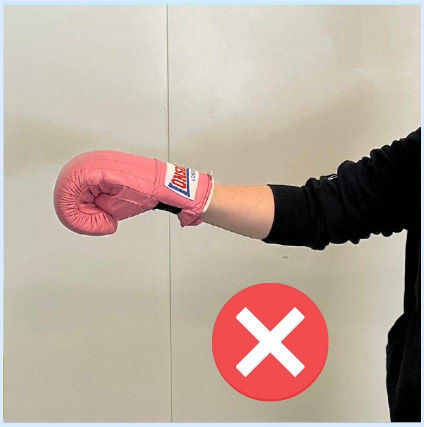

BOXING 3 - ARM POSITION

When punching forward (called a jab), make sure your hand wrist and elbow stay in a straight line to avoid injury. Correct position shown in picture

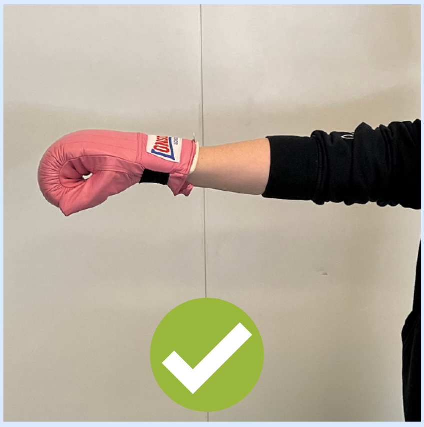

Incorrect position, which could hurt your wrist

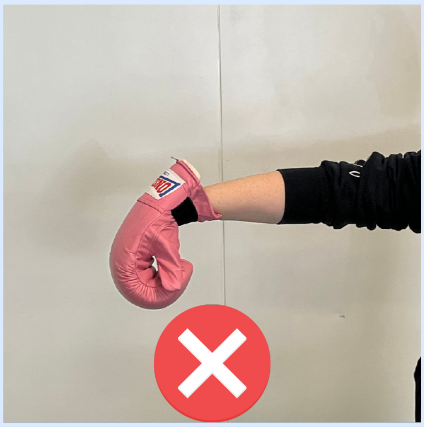

Incorrect position, which could hurt your wrist & isn't as powerful a movement

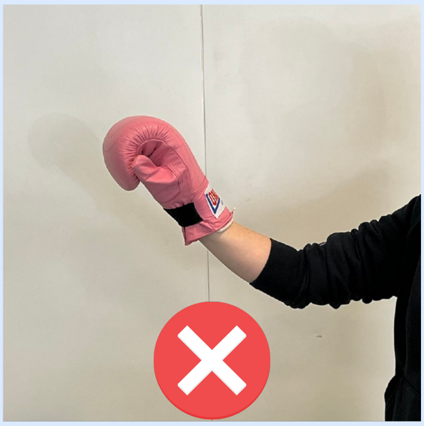

BOXING 4 - PUNCHES

This movement is called a hook punch.

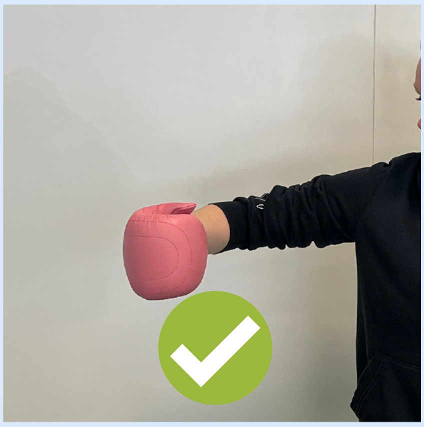

This movement is called an upper cut punch.
Start off with your knees bent then drive upwards using your legs.

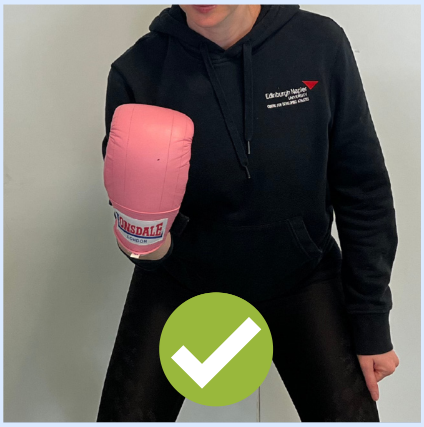

Finish the move standing upright

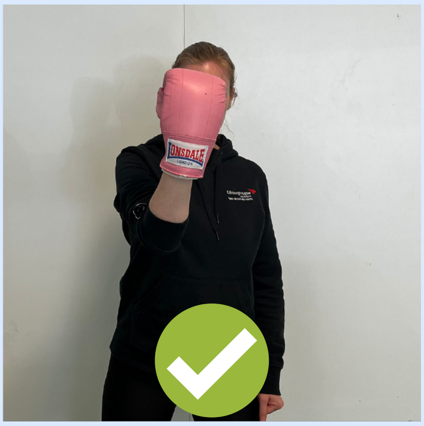

BOXING 5 - HOLDING THE FOCUS PADS

When holding the focus pads for your partner, it's important you stand a safe and strong stance.

This is how you should stand if your partner is performing jab punches.

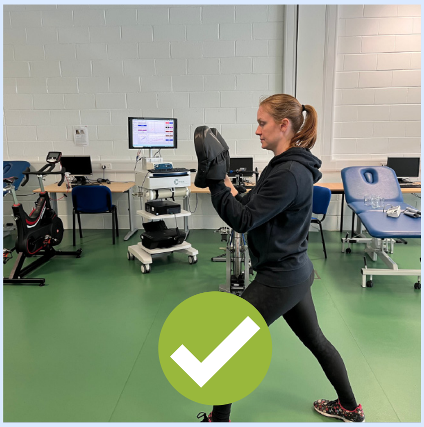

This is how you should stand if your partner is doing hook punches

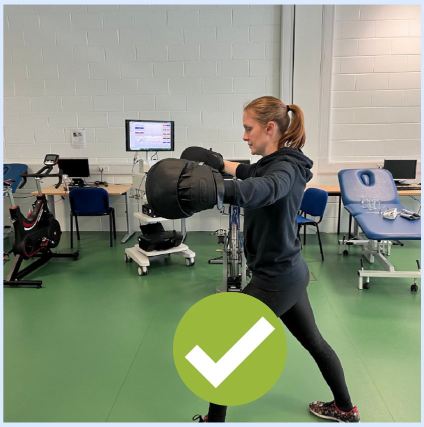

This is how you should stand is your partner is doing upper cut punches

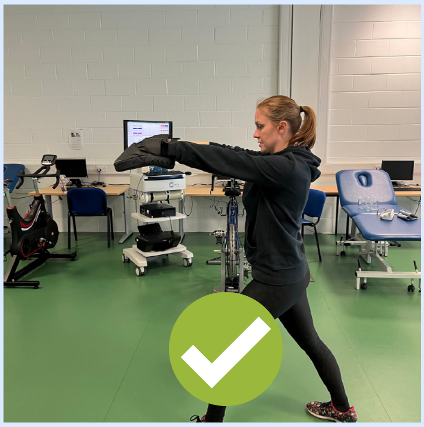

BOXING 6 - HOLDING THE FOCUS PADS

Incorrect way of holding focus pads during jabs. Pads are too close to the face and body position is too narrow.

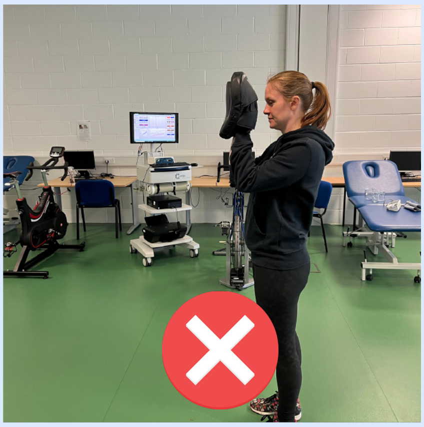

Incorrect way of holding focus pads during hooks. Focus pads are too far apart & could hurt your shoulders

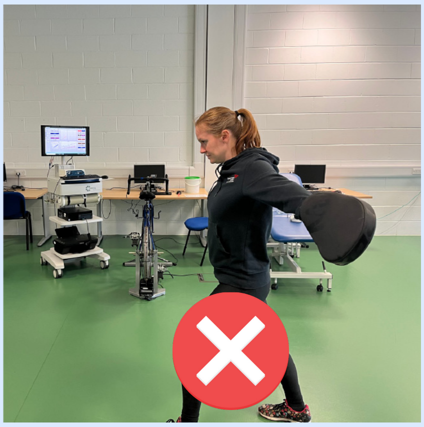

Incorrect way of holding focus pads during upper cuts. Focus pads are too high for your partner to reach

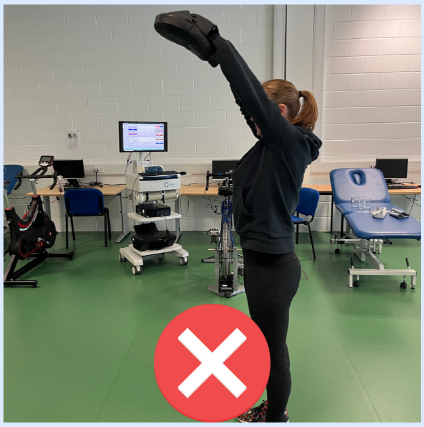

BOXING 7 - HOLDING THE FOCUS PADS

Incorrect way of holding focus pads. Focus pads are too low for your partner to reach.

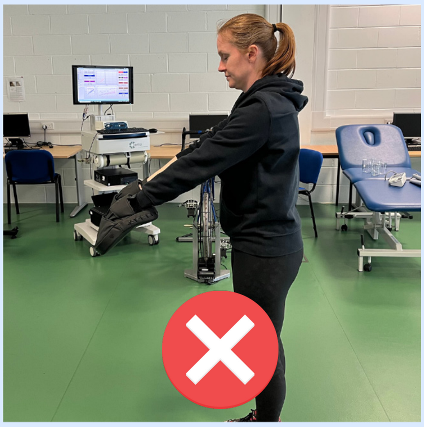

Incorrect way of holding focus pads. Pads are too close to body, which could injure you.

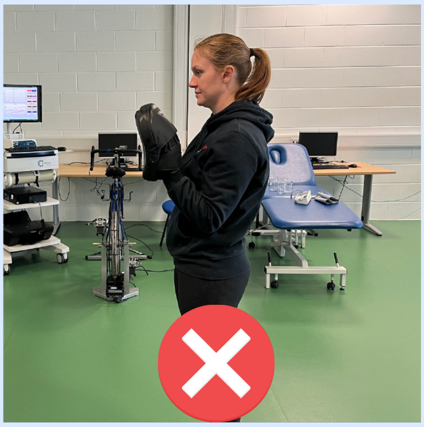

Good technique shown by puncher & partner holding focus pads

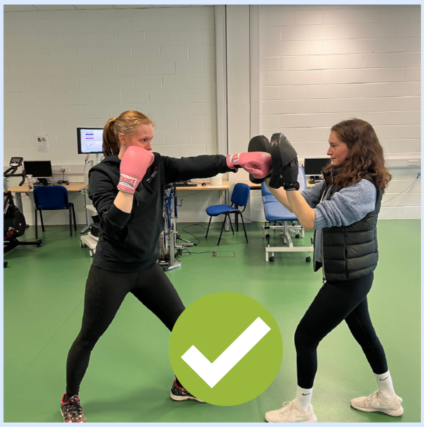

BOXING 8 - PUNCHES IN ACTION

Jab action

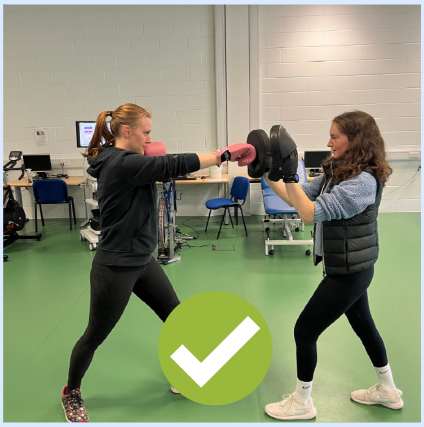

Hook action (right arm)

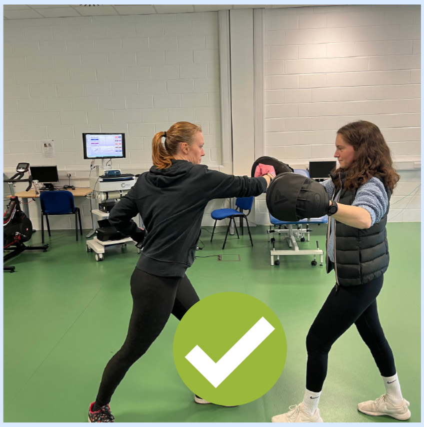

Hook action (left arm)

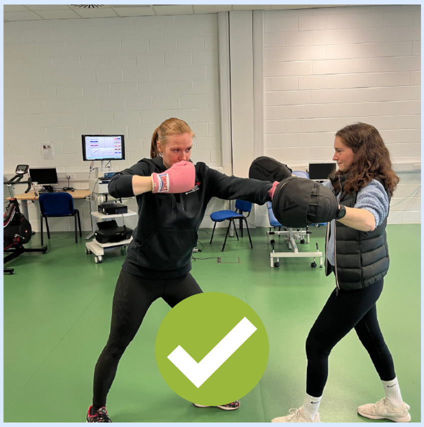

BOXING 9 - PUNCHES IN ACTION

Upper cut part 1 (right arm)

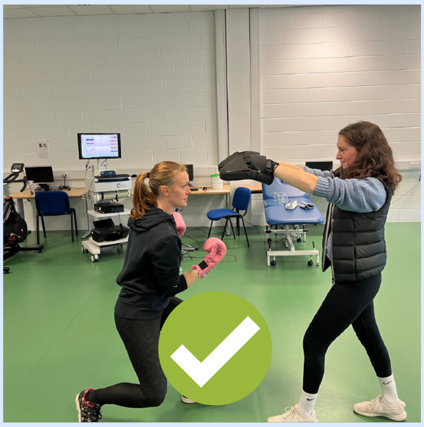

Upper cut part 2 (right arm)

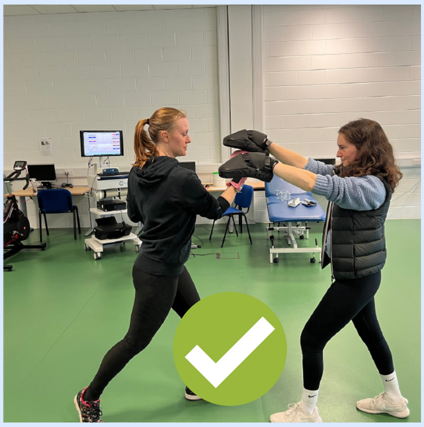

THINGS TO CONSIDER WHEN RUNNING YOUR SESSIONS

We have some key ideas that will help you to run engaging HIIT sessions that everyone will enjoy and benefit from. Try and include these SAAFE principles in your sessions:

Supportive	**SUPPORTIVE: Young Leader was supportive and promoted positive student interactions:** • Provide constructive feedback • Praise student effort and improvement • Encourage supportive behaviour among students (e.g., high fives)
Active	**ACTIVE: Session was highly active and transition time was minimal:** • Commence session quickly • Minimise talk and instruction time • Encourage students to exercise at a high intensity
Autonomous	**AUTONOMOUS: Session involved opportunities for student choice:** • Provide students with opportunities for choice (e.g., music, partner, activity) • Minimise controlling language (e.g., don't order students around)
**Fair**	**FAIR: All students provided with opportunities to experience success:** • Encourage self-comparison rather than peer-comparison • Encourage students to modify exercises to personal fitness and ability level • Treat all students equally and fairly (i.e., high expectations for all)
**Enjoyable**	**ENJOYABLE: Session was enjoyable and included a variety of activities:** • Play motivational music during exercise sessions • Provide students with a variety of HIIT workout options • Encourage students to reflect on their post exercise affect (i.e., how they are feeling)

REFLECTIONS
EXAMPLE TEMPLATE

WEEK 1	ANSWER
How do you feel the sessions went?	
Did you think anything could be improved?	
What do you think worked well?	

PLANNING YOUR SESSION TEMPLATE

Which warmup activities could you do?

Which activities does your group like the best?

What could you do in case they don't like one of the activities?
