# Supplementary material for: Feasibility of a school-based peer-led high-intensity interval training intervention: the Young Fitness Leaders project
Source: BMC Public Health. 2026 Feb 24;26:799. doi: 10.1186/s12889-026-26543-w (PMC12961876; doi:10.1186/s12889-026-26543-w)
Supplement: Supplementary file 6 — Supplementary Material 6. [file 12889_2026_26543_MOESM6_ESM.docx]

**Young fitness leaders research project**

**Focus groups**

Approx 5-7 young people per group

Order of the questions may be altered depending on the flow of the discussion

**All pictures referred to below have been removed to maintain participant’s anonymity.**

| **For all groups**, start with a welcome. Re-iterate their participation is voluntary, the discussion is confidential and they don’t have to say anything if they don’t want to, can leave without a reason etc. Ask if it’s ok to start the voice recorder, which is only there so we can concentrate on the discussion and then write it up afterwards (names will be removed from reports).  Switch on the recorder and begin an ice-breaker such as asking them to say their name and tell us their favourite food/school lesson etc. |
| --- |
| **Year 7 (peer-recipient) groups** |
| 1. Remind them of the research project: i.e. Young Leaders delivered exercise sessions to younger students. |
| 1. Outline the purpose of the focus group (e.g. to gather your opinions on how the research project went) |
| For the following questions a timeline will be used with photographs taken during the intervention used to guide the discussion. Pictures will be placed along the timeline and participants asked the following questions: |
| Place picture 1 next to Week 2 of the timeline   1. Can you tell me what you were doing in this photo?   Prompt: this is a picture showing the exercise sessions you did with the Young Leaders.   1. Did you take part in the sessions? 2. If ‘No’, were there any particular reasons for that? 3. If ‘yes’, tell me about the exercises you were doing in those picture   Prompts: how did you find being part of the sessions at the start of the project? What happened in the sessions at the start of the project? How did you feel during the exercises? |
| Place picture 2 next to week 4 – this is when the sessions transferred outside.   1. Can you tell me what you were doing in this photo?   Prompt: this is a picture showing the exercise sessions you did with the Young Leaders but OUTSIDE.   1. Did you take part in the sessions? 2. If ‘No’, were there any particular reasons for that? 3. If ‘yes’, tell me about the exercises you were doing in those picture   How did you feel about moving the sessions outside?  Prompts: preferred indoor/outdoors – why? |
| Place picture 3 next to Week 4 of the timeline   1. Can you tell me what you were doing in this photo?   Prompt: this is a picture showing the boxing sessions you did with the Young Leaders   1. Did you take part in the boxing sessions?   If ‘No’, were there any particular reasons for that?   1. If ‘yes’, tell me about the exercises you were doing in those picture   Prompts: how did you find being part of the sessions at the start of the project? What happened in the sessions at the start of the project? How did you feel during the exercises? Was the boxing different to the exercises you did at the start of the project? |
| 1. Did you attend all of the sessions?   Why? / Why not? |
| 1. Did anything make it difficult for you to take part in exercise sessions?   Prompt: embarrassment? Siblings as Young Leaders? Don’t like the exercises? Other school demands? |
| 1. What would you normally have done during those times?   [registration] |
| 1. How did you feel about doing the HIIT sessions instead? Was the time of day ok? Length of session? |
| 1. Were the sessions fun? What could have made them more enjoyable? |
| Place picture 4 next to Week 9 of the timeline   1. Can you tell me who these people are? 2. Prompt: these people are your Young Leaders that ran the exercise sessions. Can you tell me about them? Did they demonstrate the exercises and give you encouragement? Did they let you choose your exercises and music? Did your Young Leader always turn up? Did they keep the session on time? |
| 1. Would you like this programme to continue?   Why? Why not? |
| Ask if there are any final comments, then thank the participants and switch off the recorder. |
| **Year 12 and 13 Young Leaders** |
| Remind them of the research project: i.e. Young Leaders delivered exercise sessions to younger students. |
| Outline the purpose of the focus group (e.g. to gather your opinions on how the research project went) |
| 1. Do you remember taking part in a training session? 2. How did you find it? (useful or not, too fast/slow, not long enough/too long) 3. Do you remember being given a training booklet and access to an online version? 4. Did you use either of these? Why/not? |
| 1. How did you find leading of the sessions?   Prompt: Did you like / dislike doing it? |
| 1. Did you attend all of your sessions?    1. Why? / Why not? |
| 1. Did anything make it difficult for you to take part in the sessions? |
| 1. What would you normally have done during those times? |
| 1. How did you feel about doing the HIIT sessions instead? Was the time of day ok? Length of session? |
| 1. Were the sessions fun? What could have made them more enjoyable? |
| 1. Do you think your Year 7 groups enjoyed taking part? What makes you say that? |
| 1. Do you think you have gained anything from taking part? |
| 1. Is there anything that could be improved for next time? |
| Ask if there are any final comments, then thank the participants and switch off the recorder. |
| **Teachers** |
| Remind them of the research project: i.e. Young Leaders delivered exercise sessions to younger students. |
| Outline the purpose of the focus group (e.g. to gather your opinions on how the research project went) |
| 1. How do you think the project went?  Why is that? Can you explain a bit more? |
|  |
| 2. Did the project impact negatively on the pupils who were involved in any way?  Can you give an example? |
| 3. Did you seen any positive impacts on the pupils involved?  Can you give an example?  Did the school support the Young Leaders in any particular way? |
| 4. Did the project impact on the rest of the school in any way?  Positive or negative – do you have any examples? |
| 5. What do you think could be improved for next time? |
| 6. Do you think the school would continue the scheme now that the research side is finished?  Why/why not? |
| 7. How do you think we could scale up the project so more of the school can be involved? |
| 8. Has the school learnt anything that will change how it runs/supports pupils to be responsible for others? |
| Ask if there are any final comments, then thank the participants and switch off the recorder. |
